# Cytotoxicity of Root Canal Sealers and Potential Clinical Implications: A Comprehensive Systematic Review of In Vitro Studies

**DOI:** 10.3390/jcm15030973

**Published:** 2026-01-25

**Authors:** Mirko Piscopo, Angelo Aliberti, Roberta Gasparro, Gilberto Sammartino, Noemi Coppola, Pietro Ausiello

**Affiliations:** Department of Neurosciences, Reproductive and Odontostomatological Sciences, University of Naples Federico II, 80131 Naples, Italy; mirk.piscopo@studenti.unina.it (M.P.); roberta.gasparro@unina.it (R.G.); noemi.coppola@unina.it (N.C.); pietausi@unina.it (P.A.)

**Keywords:** root canal sealers, cell death, cytotoxicity, biocompatibility, root canal filling materials, MTT assay, bioactive sealers, endodontic materials, periapical tissues

## Abstract

**Background**: Root canal sealers may come into direct contact with periapical tissues, particularly in cases of apical extrusion, potentially influencing periapical healing and treatment outcomes. Cytotoxicity assessment represents a clinically relevant parameter when selecting endodontic sealers. However, evidence derived from in vitro studies remains heterogeneous and challenging to interpret from a clinical perspective. Therefore, the aim of this systematic review was to critically evaluate the in vitro cytotoxicity of all root canal sealers that have been commercially marketed over the years, excluding experimental materials, and to contextualize the findings in relation to clinically relevant experimental conditions. **Methods**: This systematic review was conducted according to PRISMA guidelines and preregistered on the Open Science Framework. PubMed, Scopus, and the Cochrane Library were searched up to 30 November 2025. In vitro studies evaluating the cytotoxicity of commercially available root canal sealers using validated cell viability or proliferation assays were included. Data extraction focused on sealer composition, setting condition, extraction protocols, exposure parameters, and cytotoxic outcomes. Due to marked methodological heterogeneity, a qualitative synthesis was performed. **Results**: Ninety-eight in vitro studies were included. All categories of root canal sealers demonstrated some degree of cytotoxicity, particularly when tested in freshly mixed conditions, at higher extract concentrations, or after prolonged exposure. Bioactive and calcium silicate-based sealers generally showed a more favorable cytotoxicity profile compared with conventional materials, especially after complete setting and at diluted concentrations, although cytotoxic effects were reported under specific experimental conditions. Resin-based sealers, including AH Plus, exhibited condition-dependent cytotoxicity, while zinc oxide–eugenol and glass ionomer sealers tended to display higher cytotoxic potential. **Conclusions**: In vitro cytotoxicity of root canal sealers varies according to material composition and experimental conditions. Bioactive sealers generally exhibit a more favorable biological profile, which may be clinically relevant in situations involving sealer extrusion or prolonged tissue contact. Standardized testing protocols and further translational studies are required to support evidence-based clinical material selection.

## 1. Introduction

Root canal treatment aims to eliminate intracanal infection and to promote long-term periapical healing. In this context, root canal sealers play a crucial role in achieving an effective seal of the root canal system; however, these materials may come into direct contact with periapical tissues, mainly in cases of apical extrusion. Such extrusion may be favored by several factors, including anatomical complexity, iatrogenic damage to the apical region, and local inflammatory or resorptive processes. Therefore, the biological behavior of endodontic sealers represents a clinically relevant aspect of material selection, as adverse tissue reactions may potentially compromise healing outcomes. Among the biological properties of dental materials, cytotoxicity has been widely investigated as a surrogate marker of biocompatibility under controlled in vitro conditions [1].

The overarching goals of endodontic treatment are the reduction of intra-radicular microbial burden, the establishment of conditions favorable for periapical tissue repair, and the long-term prevention of reinfection through a hermetic three-dimensional sealing of the canal system [2].

Endodontic therapy involves several interconnected phases, including diagnosis and case difficulty assessment, access cavity preparation, chemo-mechanical shaping, irrigation, intracanal medication when indicated, root canal obturation, definitive coronal restoration, and clinical monitoring. When biological conditions allow, current guidelines support single-visit endodontic treatment, as this approach has been shown to reduce the risk of canal recontamination. Accordingly, intracanal medicaments should not be considered a routine phase of treatment, but rather an adjunct reserved for selected clinical situations in which obturation cannot be immediately achieved, such as persistent exudation or unresolved infection. Within this framework, mechanical shaping aims to create favorable conditions for effective irrigation and disinfection of the root canal system while preserving dentinal structure and minimizing the risk of procedural errors, particularly in anatomically complex canals, while obturation seals the root canal after disinfection, ideally preventing apical leakage and recurrence of infection [3].

Gutta-percha combined with a root canal sealer remains the most widely used obturation technique. Sealers exhibit considerable chemical diversity, encompassing zinc oxide–eugenol, epoxy resin, methacrylate resin, glass ionomer, silicone-based, calcium hydroxide, and bioactive formulations [4]. According to classical criteria, an ideal sealer should demonstrate adequate adhesion to canal walls, dimensional stability, radiopacity, insolubility in biological fluids, antimicrobial properties or at least non-supportive behavior toward microbial growth, retrievability when required, and critically biocompatibility [5].

Biocompatibility holds clinical relevance because sealers may come into direct contact with periapical tissues, especially in cases of unintentional extrusion [6]. Defined as the ability of a material to trigger an appropriate and non-harmful biological response, biocompatibility represents a prerequisite for promoting periapical healing [7]. Nonetheless, all sealers display some degree of cytotoxicity, predominantly in their freshly mixed state, with toxicity typically decreasing upon setting and stabilization of the material [8]. Consequently, minimizing apical extrusion remains a fundamental clinical principle.

In vitro assays are widely employed to investigate the biological response to dental materials, providing valuable insights into their potential cytotoxic effects [9]. Various methodological approaches exist, each probing distinct cellular processes [10].

Commonly used assays include tests based on metabolic activity (e.g., resazurin, tetrazolium salts, calcein-AM fluorescence, ATP quantification, and lactate dehydrogenase (LDH) assay release) [11,12,13,14], membrane integrity (e.g., trypan blue, eosin, and erythrosin B) [15,16], lysosomal uptake (e.g., Neutral Red) [17], and DNA synthesis (e.g., Tritium-labeled (^3^H) thymidine uptake and BrdU assay) [18]. Additional methods explore cell death pathways, such as apoptosis assays (e.g., Hoechst chromatin staining, Annexin V binding, caspase activation, DNA fragmentation, Comet, and TUNEL assays) [19,20] and necrosis assays (e.g., PI uptake) [21].

Despite their relevance, in vitro cytotoxicity assessment methods differ markedly in sensitivity, specificity, exposure protocols, and cell models, generating substantial heterogeneity across studies and complicating direct comparison and synthesis of findings. Moreover, the continuously expanding range of commercially available endodontic sealers further challenges the interpretation of isolated experimental results.

In this context, the novelty of the present systematic review lies in the comprehensive inclusion of all root canal sealers that have been commercially marketed over the years, excluding experimental materials, evaluated through validated in vitro assays, combined with a structured and comparative analysis of the methodological variables that most strongly influence cytotoxicity outcomes. Specifically, this review systematically examines the impact of material condition, extract concentration, extraction time, and cell exposure duration, factors that have often been addressed inconsistently or in isolation in previous reviews.

From a clinical perspective, this condition-oriented and integrative approach allows for a more meaningful interpretation of in vitro findings and provides clinicians with an up-to-date overview of the biocompatibility profiles of endodontic sealers, supporting informed material selection aimed at promoting periapical tissue repair and long-term treatment success.

## 2. Materials and Methods

### 2.1. Protocol, Registration, and Search Strategy

A systematic review was carried out up to 30 November 2025 in accordance with the Preferred Reporting Items for Systematic Reviews and Meta-Analyses (PRISMA) statement guidelines [22,23]. The PRISMA 2020 checklist is provided in the Supplementary Materials (Table S1) [24]. A comprehensive literature search was conducted across PubMed, Scopus, and the Cochrane Library. This systematic review has been preregistered on the Open Science Framework (OSF) under the registration DOI 10.17605/OSF.IO/VHR5W. The detailed search strategy is provided in Table 1.

### 2.2. Study Design

The research question, together with the eligibility criteria, was delineated in accordance with the PICO (Population, Intervention, Comparison, and Outcomes) framework (Table 2) [25]. Accordingly, the overarching question guiding this review was articulated as follows: “*How do endodontic root canal sealers perform in terms of cell viability and cytotoxicity in experimental cell?*”.

### 2.3. Inclusion and Exclusion Criteria

Studies were eligible for inclusion if they consisted of in vitro investigations, published in English, that quantitatively evaluated the cytotoxicity of root canal sealers using validated assays measuring cell viability or proliferation. Only commercially marketed endodontic sealers were considered eligible, including materials that are no longer currently available but were previously commercialized and widely investigated in the literature. Studies were excluded if they adopted designs other than in vitro ones (e.g., in vivo or in silico designs); if data relevant to cytotoxicity assessment were incomplete, unreliable, or insufficiently reported; or if the methods used to assess cytotoxicity were unclear, inadequately described, or exclusively qualitative. Experimental or non-commercial sealers were explicitly excluded. Further exclusion criteria included studies not written in English, studies that did not employ specific viability- or proliferation-based assays, and those limited to evaluating alternative biological properties (such as antimicrobial activity), physicochemical characteristics (including bond strength, radiopacity, pH, solubility, setting or working times, and dimensional changes), or clinical performance parameters. Studies investigating materials intended for applications other than root canal obturation were not considered eligible, as such products do not fall within the definition of root canal sealers and are therefore inherently excluded by the PICO framework. This includes materials used for apexification, management of root resorption, pulpotomy, pulp capping, root-end filling, and perforation repair, as well as adhesive systems or prosthetic cementation materials.

### 2.4. Study Selection

The study selection process was conducted independently by two reviewers and structured in two sequential steps. In the first step, two authors (AA and MP) screened the titles and abstracts of all retrieved records using the Rayyan Software (web-based application, accessed in September 2025; https://www.rayyan.ai). Articles meeting the predefined inclusion criteria were marked for further assessment, whereas those not fulfilling the criteria were excluded. Any uncertainties or disagreements were resolved through consultation with a third reviewer (RG).

In the second step, the same eligibility criteria were applied to the full texts. Two authors (AA and MP) independently reviewed all articles available in full text to identify studies that specifically evaluated the in vitro cytotoxicity of root canal sealers using validated assays for cell viability or proliferation. All selected articles were subsequently subjected to a thorough and critical evaluation by three authors (AA, MP, and RG). Newly identified publications were also incorporated and assessed following the same procedure.

Any discrepancies arising during either phase of selection were resolved through collective consensus among the three reviewers. Ultimately, only full-text articles were included in the present systematic review.

### 2.5. Data Extraction

Data extraction was carried out initially by the first (AA) and second (MP) authors. All relevant information was systematically retrieved and organized into two structured tables (Table 3 and Table 4).

Table 3 encompasses the descriptive characteristics of the included studies, including first author and year of publication, sample size, method of sealer preparation, cell model employed, and the type of assay used for cytotoxicity assessment. Table 4 summarizes the primary and secondary outcomes of interest extracted from each study, specifically material conditions (setting time), extraction time, extract concentration, cell exposure duration, and the reported cytotoxic effects.

Subsequently, the third author (RG) conducted an independent verification of the compiled dataset to ensure its completeness and accuracy. Any discrepancies or uncertainties identified during this process were resolved through discussion and consensus among all authors. In instances where essential information was not clearly reported in a publication, attempts were made to contact the corresponding authors of the original studies, and any additional data obtained were incorporated into the extraction spreadsheet.

### 2.6. Quality Assessment and Critical Appraisal for the Systematic Review of Included Studies

The methodological quality of the included in vitro studies was assessed using the reporting guidelines for preclinical research on dental materials proposed by Faggion Jr. [26]. These guidelines are adapted from the original 25-item CONSORT statement, traditionally used for randomized clinical trials, to better reflect the characteristics of in vitro and preclinical experimental designs, and in the present systematic review, a subset of 15 items was applied, in line with previous methodological studies.

This checklist evaluated whether studies provided a structured abstract, a scientifically grounded introduction with clear objectives or hypotheses, and a methods section detailing the intervention, defined outcomes, sample size justification, randomization procedures, allocation concealment, blinding, and statistical analyses. It also assessed whether results were reported with effect estimates and precision, whether limitations and potential biases were discussed, and whether funding sources and protocol availability were disclosed.

For each checklist item, a *Yes* (Y) was recorded when the required information was explicitly and adequately reported. Items for which the information was entirely missing were scored as *No* (N), whereas those with incomplete, unclear, or insufficiently detailed reporting were classified as *Unclear* (U). Based on the total number of items satisfactorily fulfilled, studies were stratified into three categories of methodological risk: high risk (1–5 items), moderate risk (6–10 items), and low risk of bias (11–15 items).

## 3. Results

### 3.1. Search Results

**Figure 1:**
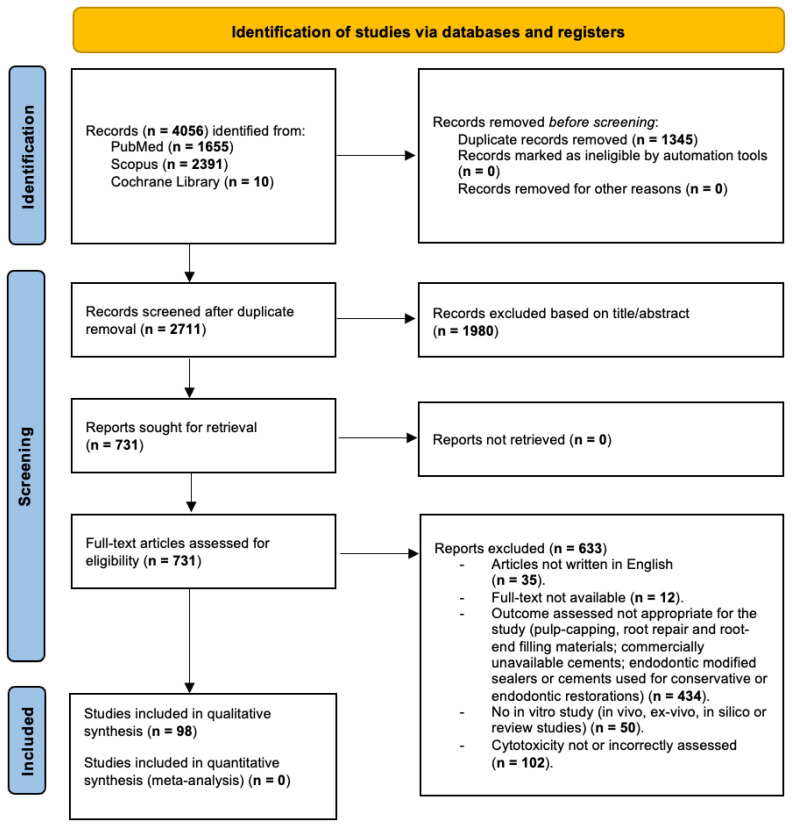
Flow diagram depicting the identification and selection of studies included in this systematic review, structured according to the Preferred Reporting Items for Systematic Reviews and Meta-Analyses (PRISMA) guidelines.

The complete workflow for article retrieval, screening, and eligibility assessment is illustrated in Figure 1.

The initial search across PubMed, Scopus, and the Cochrane Library yielded a total of 4056 records (1655 from PubMed, 2391 from Scopus, and 10 from the Cochrane Library). Before initiating the screening phase, 1345 duplicate records were identified and removed. No records were excluded through automation tools or for other reasons at this stage. Consequently, 2711 unique records proceeded to title and abstract screening.

During the screening step, 1980 records were excluded based on title and abstract evaluation because they clearly did not meet the pre-established eligibility criteria. The remaining 731 articles were retrieved for full-text assessment, and all were successfully obtained.

Full-text evaluation led to the exclusion of 633 articles for specific reasons: 35 were written in languages other than English; 12 did not provide access to the full text; 434 investigated outcomes beyond the scope of this review (including studies on pulp capping, root repair, root-end filling materials, non-commercial or unavailable cements, or modified sealers intended for non-root canal filling applications); 50 were not in vitro investigations (e.g., in vivo, ex vivo, in silico, or review articles); and 102 either did not assess cytotoxicity or used inappropriate or incorrect methodologies for its evaluation.

After completion of the screening and eligibility assessment, a total of 98 studies [27,28,29,30,31,32,33,34,35,36,37,38,39,40,41,42,43,44,45,46,47,48,49,50,51,52,53,54,55,56,57,58,59,60,61,62,63,64,65,66,67,68,69,70,71,72,73,74,75,76,77,78,79,80,81,82,83,84,85,86,87,88,89,90,91,92,93,94,95,96,97,98,99,100,101,102,103,104,105,106,107,108,109,110,111,112,113,114,115,116,117,118,119,120,121,122,123,124] met all the inclusion criteria and were incorporated into the qualitative synthesis. None of the included studies provided data suitable for quantitative synthesis, and therefore no meta-analysis was performed.

The endodontic sealers tested in vitro in the studies included in this review are grouped into their respective categories in Table S2.

### 3.2. Summary of Methodological Characteristics of the Included Studies

The methodological characteristics of the included in vitro studies are presented in Table 3.

**Table 3:** Overview of in vitro studies included in the systematic review evaluating the cytotoxicity of root canal sealers. The table summarizes, for each study, the year of publication, tested sealers and control groups, sample size and number of independent experiments, method of sealer preparation, cell models employed, and cytotoxicity assays used.

Year	Study	Tested Sealers and Controls	Sample Size and/or (Independent Experiments)	Test Procedure	Cell Model	Assay(s)
2025	Chen Z. et al. [27]	CRoot SP, i-MTA SP, iRoot SP, and NeoSEALER Flo Negative control = medium	n = 5	Test on extracts	BMMs	CCK-8
	Gaafar S. S. et al. [28]	CeraSeal and NeoSEALER Flo Positive control = AH Plus	n = 10 (3 times)	Test on extracts	hGF	MTT assay
	Kwan D.C.Y. et al. [29]	AH Plus, AH Plus Bioceramic, CRoot SP, and iRoot SP Negative control = medium	n = 3 (3 times)	Test on extracts	MC3T3-E1	CCK-8
	Nashibi S. et al. [30]	MTA Angelus and AGM MTA Negative control = medium	n = 3	Test on extracts	hDPSC	MTT assay
	Pitzschk M. A. L. R. et al. [31]	AH Plus Jet, Bio-C Sealer, and EndoSequence BC HiFlow Negative control = medium	n = 15 (3 times)	Test on extracts	SAOS-2	MTT assay
	Ramos R.F. et al. [32]	Bio-C Sealer, Bio-C Sealer ion +, and AH Plus Negative control = medium	n = 3 (3 times)	Test on extracts	hPDLSCs	MTT assay
	Santiago M.C. et al. [33]	AH Plus Bioceramic, Bio-C Sealer, NEOMTA Plus, and MTA Fillapex Negative control = medium	n = 9 (3 times)	Test by indirect contact (filter diffusion)	hDPC SAOS-2	MTT assay
	Wang Z. et al. [34]	iRoot SP and NeoSEALER Flo Negative control = medium	n = 5 (3 times)	Test on extracts	BMMs	CCK-8
	Ye Y. et al. [35]	EndoSequence BC Sealer, AH Plus Bioceramic, and AH Plus Jjet Negative control = medium	n = 15 (3 times)	Test on extracts	hPDLSCs	MTT assay
2024	Chen J. H. et al. [36]	AH Plus Jet, AH Plus Bioceramic, BioRoot RCS, and BioRoot Flow Negative control = HBSS	n = 3 (3 times)	Test on extracts	SAOS-2	Trypan Blue Dye exclusion assay
	Çelebi Keskin İ. S. et al. [37]	Sealapex, Apexit Plus, AH Plus, TotalFill BC, and MTA Fillapex Negative control = medium	n = 6 (3 times)	Test on extracts	hPDLF	MTT assay
	López-García S. et al. [38]	Neosealer Flo, AH Plus Bioceramic, and TotalFill BC Negative control = medium	(3 times)	Test on extracts	hPDLSCs	MTT assay
	Sanz J. L et al. [39]	BioRoot Flow, AH Plus Bioceramic, and AH Plus Negative control = medium	n = 3 (3 times)	Test on extracts	hPDLSCs	MTT assay
	Zhou G. et al. [40]	CRoot SP and iRoot SP AH Plus Negative control = medium	(3 times)	Test on extracts	rSCAPs	CCK-8 assay
2023	Kandemir Demirci G. et al. [41]	AH Plus Bioceramic, TotalFill BC, AH Plus, and AH Plus Jet Positive control = Doxorubicin	n = 12 (3 times)	Test by direct contact Test on extracts	SAOS-2 THP-1 hPDLF	MTT assay
	Melo A. P. et al. [42]	BioRoot RCS, Bio-C Sealer, and Sealer Plus Bioceramic Negative control = medium	n = 4 (3 times)	Test on extracts	RAW 264.7	MTT assay
	Nguyen L. C. H. et al. [43]	CeraSeal and AH Plus Negative control = medium	(3 times)	Test on extracts	hSCAPs	Alamar Blue assay
	Yan Y. et al. [44]	EndoSequence BC HIFlow, iRoot SP, and AH Plus Negative control = medium	(3 times)	Test on extracts	hSCAPs	CCK-8 assay
2022	Oliveira P. Y. et al. [45]	Endofill, Sealer 26, White MTA, and Pulp Canal Sealer Negative control = sterilized polyethylene capillary tubes	(3 times)	Test by direct contact	HDPSC	MTT assay Trypan Blue Dye exclusion assay
	Pedrosa M. D. S. et al. [46]	Bio-C Sealer, MTA Fillapex, and Cimmo HP Negative control = medium	n = 9	Test on extracts	hPDLSCs	MTT assay
	Rosatto C. M. P. et al. [47]	EndoSequence BC, Bio-C Sealer, and Sealer Plus Bioceramic Negative control = medium	n = 6	Test on extracts	BMMs	MTT assay
	Sanz J. L. et al. [48]	AH Plus Bioceramic, AH Plus, and EndoSequence BC Negative control = medium	n = 3 (3 times)	Test on extracts	hPDLSCs	MTT assay
	Tomokiyo A. et al. [49]	SuperBond sealer, Nishika Canal Sealer BG, and GuttaFlow 2 Negative control = medium	n = 4 (4 times)	Test by direct contact	hPDLSCs	Cell counting MTT assay
	Wuersching S. N. et al. [50]	AH Plus, GuttaFlow bioseal, BioRoot RCS, and TotalFill BC Negative control = medium	(3 times)	Test on extracts	hPDLF hOB	WST-1 assay
2021	Dhopavkar V. V. et al. [51]	AH Plus, MTA Filapex, and GuttaFlow2 Negative control = medium	n = 15 (3 times)	Test by direct contact	hPDLF	MTT assay
	Erdogan H. et al. [52]	AHPlus, MTA-Fillapex, and iRoot SP Negative control = medium	n = 6 (3 times)	Test on extracts	hPDLF	XTT assay
	Kan M. T. et al. [53]	AH Plus, GuttaFlow 2, Dia-ProSeal, and Pulpdent Root Canal Sealer Negative control = medium	n = 6 (3 times)	Test on extracts	L929	Alamar Blue assay
	Park M. G. et al. [54]	AH Plus, BrightEndo MTA, CeraSeal, EndoSeal TCS, and One-Fil Negative control = medium	n/s	Test on extracts	hPDLF	MTT assay
	Pawinska M. et al. [55]	Endomethasone N, Roeko Seal Automix, RealSeal, and Sealapex Negative control = medium	n = 2 (2 times)	Test by indirect contact (filter diffusion)	hPDLF	Annexin V-FITC/PI assay
	Pedrosa M. D. S. et al. [56]	Bio-C Sealer, MTA Fillapex, and Cimmo HP Negative control = medium	(3 times)	Test on extracts	hPDLSCs	MTT assay
	Zordan-Bronzel C. L. et al. [57]	Sealer Plus Bioceramic, TotalFill BC, and AH Plus Negative control = medium Positive control = 20% dimethyl sulfoxide	n = 3 (3 times)	Test on extracts	hOB SAOS-2	MTT assay Neutral Red assay
2020	Chen B. et al. [58]	EndoSequence BC HiFlow and EndoSequence BC Negative control = medium	n/s	Test on extracts	hPDLF	CCK-8
	López-García S. et al. [59]	Endoseal MTA, Ceraseal, and EndoSequence BC Negative control = medium	n = 3	Test on extracts	hPDLSCs	MTT assay
	Rodríguez-Lozano F. J. et al. [60]	EndoSequence BC HiFlow, EndoSequence BC, and AH Plus Negative control = medium	(3 times)	Test on extracts	hPDLSCs	MTT assay
2019	Jeanneau C. et al. [61]	BioRoot RCS and Pulp Canal Sealer Negative control = medium	n = 3 (3 times)	Test on extracts	LPS-stimulated hPDLF	MTT assay
	Jung S. et al. [62]	AH Plus, MTA Fillapex, BioRoot RCS, and Pulp Canal Sealer Negative control = medium	(3 times)	Test on extracts	hPDLCs	MTT Living cell count LIVE/DEAD assay
	Lee B. N. et al. [63]	AH Plus, MTA Fillapex, and EndoSequence BC Negative control = medium	n = 3 (2 times)	Test on extracts	MC3T3-E1	WST-1 assay
	Lee J. K. et al. [64]	AH Plus, AD Seal, EndoSeal MTA, Nano-Ceramic Sealer, and Wellroot ST Negative control = n/s	n/s	Test on extracts	hPDLSCs	MTT assay
	López-García S. et al. [65]	Bio-C Sealer, TotalFill BC, and AH Plus Negative control = medium	n = 30 (3 times)	Test on extracts	hPDLSCs	MTT assay
	Rodríguez-Lozano F. J. et al. [66]	AH Plus, MTA Fillapex, GuttaFlow 2, and GuttaFlow Bioseal Negative control = plastic	n = 5 (2 times)	Test on extracts	hPDLSCs	Annexin-V/7-AAD double-staining assay
	Seo D. G. et al. [67]	AH Plus, EndoSequence BC, BioRoot RCS, and Endoseal MTA Negative control = hDPSCs cultured without experimental disks	(4 times)	Test by indirect contact (filter diffusion)	hDPSC	MTT assay
2018	Alsubait S. A. et al. [68]	BioRoot RCS, Endosequence BC, and AH Plus Jet Negative control = medium	n = 3 (3 times)	Test on extracts	hMSCs	Alamar Blue assay
	Jung S. et al. [69]	AH Plus, Pulp Canal Sealer, MTA Fillapex, and BioRoot RCS Negative control = medium	(3 times)	Test on extracts	hOB	MTT Living cell count LIVE/DEAD assay LDH assay
	Martinho F. C. et al. [70]	Real Seal, AH Plus, EndoRez, and Apexit Negative control = medium	n/s	Test on extracts	hDPC	MTT assay
	Szczurko G. et al. [71]	AH Plus Jet, Apexit Plus, MTA Fillapex, GuttaFlow, MetaSEAL Soft, and Tubli-Seal Negative control = medium	n = 4	Test by indirect contact (filter diffusion)	hPDLF	MTT assay
	Taraslia V. et al. [72]	MTA Fillapex, GuttaFlow 2, EndoSequence BC, Bioroot RCS, AH Plus, and Roth’s 801 Negative control = Transwell system without material	n = 5 (5 times)	Test by indirect contact (filter diffusion)	hPDLCs	Trypan Blue Dye exclusion assay
	Vouzara T. et al. [73]	SimpliSeal, MTA Fillapex, and BioRoot RCS Negative control = medium	n = 6 (2 times)	Test on extracts	NIH/3T3	Sulforhodamine B assay
2017	Arun S. et al. [74]	Tubliseal, AH Plus, Sealapex, and EndoREZ Negative control = medium	(3 times)	Test by direct contact	L929	MTT assay
	Cintra L.T.A. et al. [75]	Sealer Plus, AH Plus, Endofill, and SimpliSeal Negative control = medium	n = 3	Test on extracts	L929	MTT assay
	Collado-González M. et al. [76]	GuttaFlow Bioseal, Guttaflow 2, MTA Fillapex, and AH Plus Negative control = medium	n = 5 (2 times)	Test on extracts	hPDLSCs	MTT assay
	Collado-González M. et al. [77]	BioRoot RCS, Endoseal MTA, and Nano-ceramic Sealer Negative control = medium	n = 5	Test on extracts	hPDLSCs	MTT assay
	Lv F. et al. [78]	iRoot FS, iRoot BP Plus, and ProRoot MTA Negative control = medium	(3 times)	Test on extracts	MC3T3-E1	CCK-8 assay
	Rodríguez-Lozano F.J. et al. [79]	MTA Fillapex, TotalFill BC, and AH Plus Negative control = medium	n = 5	Test on extracts	hPDLSCs	MTT assay
2016	Silva E. J. et al. [80]	AH plus, Endomethasone N, EndoSequence BC, MTA Fillapex, and Pulp Canal Sealer EWT Negative control = medium	n = 10	Test on extracts	Monolayer and 3D cell culture of Balb/c 3T3 fibroblasts	MTT assay
	Silva E. J. et al. [81]	AH Plus and MTA Fillapex Negative control = medium	n = 9	Test on extracts	hOB	XTT assay Neutral Red assay Crystal violet dye elution
	Suciu I. et al. [82]	MTA Fillapex, AH Plus, and Acroseal Negative control = plastic surface	n = 3	Test by direct contact	hOB DF-MSC	Alamar blue assay
2015	Dimitrova-Nakov S. et al. [83]	BioRoot RCS and Pulp Canal Sealer Negative control = untreated cells	(3 times)	Test by direct contact	A4 Pulp cells	Trypan Blue exclusion assay
	Mestieri L.B. et al. [84]	MTA Plus, MTA Fillapex, and FillCanal Negative control = medium	n = 3 (3 times)	Test on extracts	hDPC	MTT assay Neutral Red assay
2014	Camargo C.H. et al. [85]	AH Plus, RoekoSeal, and EndoRez Negative control = medium	n = 4 (3 times)	Test on extracts	V79	MTT assay
	Chang S.W. et al. [86]	Sealapex, Apatite root sealer, MTA Fillapex, and iRoot SP Negative control = medium with or without osteogenic supplements	n = 4 (3 times)	Test by direct contact	hPDLCs	MTT assay
	Cotti E. et al. [87]	RealSeal XT and AH Plus Jet Negative control = Untreated cells	N = 4 (3 times)	Test by direct contact	L929	Neutral Red assay MTT assay
	Manda P. et al. [88]	AH Plus, ProRoot MTA, RealSeal, and GuttaFlow 2 Negative control = medium	n = 5	Test on extracts	hGF	CCK-8
2013	Guven E.P. et al. [89]	iRoot SP, MTA Fillapex, and AH Plus Jet Negative control = medium	n = 6	Test by indirect contact (filter diffusion)	hTGSCs	MTS assay
	Kim T.G. et al. [90]	AH Plus Negative control = medium	(3 times)	Test on extracts	MC3T3-E1	MTT assay
2012	Bin C.V. et al. [91]	White MTA, MTA Fillapex, and AH Plus Negative control = medium	n = 4 (3 times)	Test on extracts	V79	MTT assay
	Salles L.P. et al. [92]	MTA Fillapex, Epiphany SE, and EndoFill Negative control = Transwell system without material	n = 2 (3 times)	Test by indirect contact (filter diffusion)	SAOS-2	MTT assay
	Scelza M.Z. et al. [93]	Real Seal SE, AH Plus, GuttaFlow, Sealapex, Roth 801, and ThermaSeal Plus Negative control = medium	N = 3 (2 times)	Test on extracts	hGF	MTT assay
	Shon W.J. et al. [94]	Apatite Root Sealer Type I, Apatite Root Sealer Type III, CAPSEAL I, CAPSEAL II, and Pulp Canal Sealer EWT Negative control = Transwell system without material	n = 6	Test by indirect contact (filter diffusion)	MG63	MTT assay
	Van Landuyt K.L. et al. [95]	EndoRez, RealSeal, AH Plus Jet, and Calcicur Negative control = medium	n = 4 (4 times)	Test on extracts	hGF	XTT assay
2011	Loushine B.A. et al. [96]	EndoSequence BC, AH Plus, and Pulp Canal Sealer EWT Negative control = Teflon	n = 6	Test by indirect contact (filter diffusion)	MC3T3-E1	MTT assay
	Zoufan K. et al. [97]	AH Plus, Tubli-Seal Xpress, GuttaFlow, and Endosequence BC Negative control = medium	(3 times)	Test on extracts	L929	MTT assay
2010	Al-Hiyasat A.S. et al. [98]	EndoRez, AH Plus, Epiphany, and MetaSeal Negative control = medium	n/s	Test on extracts	Balb/c 3T3 fibroblasts	MTT assay
	Bae W.J. et al. [99]	AH 26, Sankin apatite root canal sealer, Pulp Canal Sealer EWT, CAPSEAL I, and CAPSEAL II Negative control = medium	n = 5 (3 times)	Test on extracts	hPDLCs	MTT assay
	Ghanaati S. et al. [100]	AH Plus and Gutta Flow Negative control = medium	n = 3	Test by direct contact	hPDLF	Alamar Blue assay Toxilight Kit assay
	Huang F.M. et al. [101]	AH 26, Canals, and N2 Negative control = medium	n = 3 (3 times)	Test on extracts	U2OS	Alamar blue assay
	Yu M.K. et al. [102]	AH 26 Negative control = medium	(3 times)	Test on extracts	MC3T3-E1	MTT assay
	Zhang W. et al. [103]	IRoot SP and AH Plus Negative control = medium	n = 6 (2 times)	Test on extracts	MG63	MTT assay
2009	Ames J.M. et al. [104]	EndoREZ, RealSeal, MetaSEAL, RealSeal SE, and Pulp Canal Sealer Negative control = Teflon	n = 6	Test by direct contact	ROS 17/12.8	MTT assay
	Correa G.T. et al. [105]	AH Plus, Fill Canal, and L&C Negative control = medium	n = 3	Test on extracts	THP-1	Trypan Blue Dye exclusion assay
	Donadio M et al. [106]	Activ GP, RealSeal, AH 26, and Kerr Sealer Negative control = medium	(3 times)	Test on extracts	L929	MTT assay
	Gambarini G. et al. [107]	Epiphany SE, Epiphany, and Pulp Canal Sealer Negative control = medium	n = 6	Test on extracts	3T3 fibroblast	Neutral Red assay
	Huang F.M. et al. [108]	AH 26, Canals, and N2 Negative control = medium	n = 3 (3 times)	Test on extracts	U2OS	Propidium iodide assay
2008	Huang F.M. et al. [109]	AH26, Canals, and N2 Negative control = medium	n = 3 (3 times)	Test on extracts	U2OS	Hoechst 33258 fluorescence
	Lodiene G. et al. [110]	AH Plus, EndoREZ, RoekoSeal, Automix, and Epiphany Negative control = medium (extract) and PTFE (filter)	Indirect with filter: EndoREZ and AH Plus n = 6 RoekoSeal Automix and Epiphany n = 9 Indirect with extract: n = 16	Test on extracts Test by indirect contact (filter diffusion)	L929	MTT assay Nitro-blue tetrazolium
	Pinna L. et al. [111]	MetaSEAL, AH Plus Jet, and Pulp Canal Sealer Negative control = Teflon Positive control = PMMA	n = 6	Test by direct contact	ROS 17/12.8	MTT assay
	Valois C.R. et al. [112]	AH Plus, Endofill, and Sealer 26 Negative control = medium	n = 6 (2 times)	Test on extracts	3T3 fibroblast	MTT assay
2007	Eldeniz A.U. et al. [113]	Epiphany, EndoREZ, RC Sealer, Acroseal, GuttaFlow, AH Plus, Apexit, and RoekoSeal Negative control = medium	n = 6 (2 times)	Test on extracts	hGF L929	MTT assay
	Merdad K. et al. [114]	Epiphany and AH Plus Negative control = filters with cells and no sealer	n = 10 (3 times)	Test by direct contact Test by indirect contact (filter diffusion)	HeLa	Nitro Blue Tetrazolium dye assay
2005	Miletic I. et al. [115]	Roekoseal Automix and AH Plus Negative control = untreated cells	n = 4 (2 times)	Test by direct contact	HeLa L929	Nigrosin Dye assay
2004	Bouillaguet S. et al. [116]	Pulp Canal Sealer, Roeko Seal Automix, Top Seal, and Endo REZ Negative = Teflon	n = 4	Test by direct contact	Balb/c 3T3 fibroblasts	MTT assay
	Huang T.H. et al. [117]	Sealapex, Canals, and AH Plus Negative control = medium	n = 8 (3 times)	Test on extracts	OC2	MTT assay
2002	Huang T.H. et al. [118]	AH Plus and AH 26 Negative control = medium	n = 5	Test on extracts	Rat cerebral astrocytes	MTT assay
	Schwarze T. et al. [119]	AH Plus, Apexit, Endomethasone, Ketac Endo, and N2 Negative control = medium	n = 6 (3 times)	Test on extracts	3T3 fibroblast hPDLF	XTT assay
2001	Huang T.H. et al. [120]	Canals, Sealapex, AH 26, and AH Plus Negative control = medium	n = 5 (3 times)	Test on extracts	Rat hepatocytes	LDH leakage assay
2000	Azar N.G. et al. [121]	AH 26, AH Plus, and Zinc Oxide Eugenol Negative control = medium	n = 2 (4 to 8 times)	Test on extracts	hGF	Neutral Red assay
	Huang T.H. et al. [122]	AH 26 and AH Plus Negative control = medium	(3 times)	Test by direct contact	Rat hepatocytes	LDH leakage assay
1999	Telli C. et al. [123]	Sankin apatite root canal sealers I, Sankin apatite root canal sealers II, Sankin apatite root canal sealers III, Calciobiotic Root Canal Sealer, Ketac Endo, AH26, and Endomethasone Negative control = medium	n = 4	Test on extracts	L929	MTT assay
1997	Beltes P. et al. [124]	Ketac Endo and Endion Negative control = medium	n = 4 (2 times)	Test by direct contact	BHK21/C13	Trypan Blue Dye exclusion assay

**Abbreviations**: (n) represents sample size. Cell lines: hDPSC, human dental pulp stem cell; hDPC, human dental pulp cell; SAOS-2. human osteoblastic cell; HeLa, HeLa, human cervical cancer cell; hGF, human gingival fibroblast; hMSCs, immortalized human bone marrow-derived mesenchymal stem cells; hPDLF, human periodontal ligament fibroblast; hOB, human osteoblast; DF-MSC, dental follicle mesenchymal stem cell; hPDLCs, human periodontal ligament cells; hPDLSCs, human periodontal ligament stem cells; hSCAPs, human apical papilla stem cells; hTGSCs, human tooth germ stem cells; OC2, human oral cancer cell line; THP-1, human monocytic cells; U2OS, human osteoblastic cell line; MG63, human osteoblast-like cells; BMMs, bone marrow-derived monocyte–macrophages; rSCAPs, rat apical papilla stem cells; RAW 264.7, RAW 264.7 mouse macrophages; L929, L929 mouse fibroblasts; MC3T3-E1, mouse osteoblast-like cells; NIH/3T3, NIH/3T3 mouse fibroblasts; V79, Chinese hamster fibroblasts; ROS 17/12.8, ROS 17/12.8 rat osteosarcoma cells; BHK21/C13, Baby Hamster Kidney; EWT, Extended Working Time; BC, bioceramic.

Among the 98 included studies [27,28,29,30,31,32,33,34,35,36,37,38,39,40,41,42,43,44,45,46,47,48,49,50,51,52,53,54,55,56,57,58,59,60,61,62,63,64,65,66,67,68,69,70,71,72,73,74,75,76,77,78,79,80,81,82,83,84,85,86,87,88,89,90,91,92,93,94,95,96,97,98,99,100,101,102,103,104,105,106,107,108,109,110,111,112,113,114,115,116,117,118,119,120,121,122,123,124], substantial methodological heterogeneity was observed in the experimental setup used to evaluate cytotoxicity. Most investigations employed tests on extracts, while a smaller proportion used tests by direct contact between cells and the material; specifically, tests by direct contact were adopted in 15 studies [45,49,51,74,82,83,86,87,100,104,111,115,116,122,124], while tests on extracts (eluates) represented the predominant approach, used in 71 studies [27,28,29,30,31,32,34,35,36,37,38,39,40,42,43,44,46,47,48,50,52,53,54,56,57,58,59,60,61,62,63,64,65,66,68,69,70,73,75,76,77,78,79,80,81,84,85,88,90,91,93,95,97,98,99,101,102,103,105,106,107,108,109,112,113,117,118,119,120,121,123]. Only nine studies used tests by indirect contact (filter diffusion) [33,55,67,71,72,89,92,94,96]. A few studies combined approaches to improve methodological robustness: one study employed both test by direct contact and test on extracts [41], one study used both test on extracts and test by indirect contact (filter diffusion) [110], while another one combined test by direct contact with test by indirect contact (filter diffusion) [114].

Regarding the cellular models used for the assessment of cell viability, a broad range of human and animal cell lines were used, reflecting the diversity of biological environments potentially affected by root canal sealers. These included stem cells, fibroblasts, osteoblastic lineages, cancer-derived lines, and immune cells, such as human dental pulp stem cells (hDPSCs) [30,45,67], human dental pulp cells (hDPCs) [33,70,84], human osteoblastic cells (SAOS-2) [31,33,36,41,57,92], HeLa human cervical cancer cells (HeLa) [114,115], human gingival fibroblasts (hGFs) [28,88,93,95,113,121], immortalized human bone marrow-derived mesenchymal stem cells (hMSCs) (68), human periodontal ligament fibroblasts (hPDLFs) [37,41,50,51,52,54,55,58,61,71,100,119], human osteoblasts (hOBs) [50,57,69,81,82], dental follicle mesenchymal stem cells (DF-MSCs) (82), human periodontal ligament cells (hPDLCs) [62,72,86,99], human periodontal ligament stem cells (hPDLSCs) [32,35,38,39,46,48,49,56,59,60,64,65,66,76,77,79], human apical papilla stem cells (hSCAPs) [43,44], human tooth germ stem cells (hTGSCs) [89], a human oral cancer cell line (OC2) [117], THP-1 human monocytic cells (THP-1) [41,105], the U2OS human osteoblastic cell line (U2OS) [101,108,109], human osteoblast-like cells (MG63) [94,103], and bone marrow-derived monocyte–macrophages (BMMs) [27,34,47]. Several studies used rodent-derived or immortalized lines, including rat apical papilla stem cells (rSCAPs) [40], RAW 264.7 mouse macrophages (RAW 264.7), [42] L929 mouse fibroblasts (L929) [53,74,75,87,97,106,110,113,123], MC3T3-E1 mouse osteoblast-like cells (MC3T3-E1) [29,63,78,90,96,102], NIH/3T3 mouse fibroblasts (NIH/3T3) [73], Balb/c 3T3 fibroblasts [80,98,116], A4 pulp cells [83], V79 Chinese hamster fibroblasts (V79) [85,91], ROS 17/12.8 rat osteosarcoma cells (ROS 17/12.8) [104,111], 3T3 fibroblasts [107,112,119], rat cerebral astrocytes [118], rat hepatocytes [120,122], and BHK21/C13 baby hamster kidney cells (BHK21/C13) [124]. This variability in cellular models contributes to differences in cytotoxicity outcomes across studies.

As regards, on the other hand, the techniques used to assess cell viability, most studies relied on metabolic activity assays, particularly tetrazolium-based tests.

In particular, 61 studies used the 3-[4,5-dimethylthiazol-2-yl]-2,5-diphenyltetrazolium bromide (MTT) assay [28,30,31,32,33,35,37,38,39,41,42,45,46,47,48,49,51,54,56,57,59,60,61,62,64,65,67,69,70,71,74,75,76,77,79,80,84,85,86,87,90,91,92,93,94,96,97,98,99,102,103,104,106,110,111,112,113,116,117,118,123]. A total of four studies used the 2,3-bis-(2-methoxy-4-nitro-5-sulfophenyl)-2H-tetrazolium-5-carboxanilide (XTT) assay [52,81,95,119]; six used the Alamar Blue assay [43,53,68,82,100,101]; eight used the Cell Counting Kit 8 (CCK-8/WST-8) assay [27,29,34,40,44,58,78,88]; two used the Water-Soluble Tetrazolium Salt-1 (WST-1) assay [50,63]; and one study used the 3-(4,5-dimethylthiazol-2-yl)-5-(3-carboxymethoxyphenyl)-2-(4-sulfophenyl)-2H-tetrazolium (MTS) assay [89]. Other studies used the Sulforhodamine B assay, Trypan Blue dye exclusion, Neutral Red uptake, and additional tests [36,45,49,57,72,73,81,83,84,87,89,105,107,108,109,114,115,121,124]. Moreover, eight studies employed more than one technique for cell viability assessment [45,49,57,62,69,81,84,110].

Furthermore, data showed that the cytotoxicity of root canal sealers varied significantly depending on their state of polymerization or setting state. Only fresh materials were analyzed in 18 studies [61,68,74,80,87,90,93,95,98,100,102,105,109,112,118,120,121,122], while only set materials after different incubation periods were evaluated in 57 studies [27,28,29,31,32,33,34,36,39,40,41,42,43,44,45,46,47,48,49,50,51,56,57,58,59,60,63,65,66,67,70,73,75,76,77,78,79,82,83,84,86,89,92,94,96,99,101,103,104,107,108,111,115,123,124]. Among these, 24 studies used a standardized 24 h setting period. Finally, both fresh and set conditions were tested in 22 studies [30,35,52,53,54,55,62,64,71,72,81,85,88,91,97,106,110,113,114,116,117,119]. This allowed several studies to directly compare how setting influences cytotoxicity.

Among studies using the extract method, extraction time was a key variable. The 24 h extraction period was the most common, used in 62 studies [12,28,30,31,32,35,36,37,38,39,41,42,43,44,47,48,50,52,53,54,57,58,59,60,61,62,64,65,66,68,69,70,73,76,77,79,80,81,84,85,88,90,91,93,95,97,101,102,103,105,106,107,108,109,110,112,113,117,118,119,120,121]. Multiple extraction times were evaluated in 11 studies [36,50,54,64,73,88,93,97,102,106,121], enabling assessment of time-dependent elution patterns.

To determine dose–response relationships, 58 studies tested more than one extract concentration [27,28,29,30,31,32,34,35,37,38,39,40,41,42,43,44,46,47,48,50,52,56,57,58,59,60,62,63,65,68,69,70,73,75,76,77,78,79,84,85,88,91,95,97,98,99,101,103,105,106,108,109,112,117,118,120,122,123]. This allowed for the identification of concentration-dependent cytotoxic effects, which are critical for interpreting material behavior in clinical contexts.

In conclusion, moreover, most studies analyzed the effect of different cell exposure times, reflecting the dynamic interaction between sealers and biological tissues. A total of 55 studies modulated exposure duration to evaluate its impact on cell viability [27,28,29,30,31,34,35,37,38,39,40,41,43,44,45,46,47,48,51,53,54,56,57,58,59,60,61,62,64,65,67,68,69,73,75,76,77,79,82,83,86,87,89,92,94,96,99,100,102,104,111,116,122,124]. Time-dependent variations were widely observed, reinforcing the importance of temporal factors in cytotoxicity outcomes.

### 3.3. In Vitro Cytotoxicity of Root Canal Sealers

In general, all categories of endodontic sealers showed some degree of toxicity (Table 4). To provide a more structured interpretation of these results, the cytotoxicity findings were organized according to the specific material class of each sealer, based on their intrinsic physicochemical characteristics, as detailed in Table S2.

**Table 4:** Experimental conditions and main cytotoxic outcomes of the included in vitro studies. For each investigation, the table reports the tested sealers, material condition (fresh or set), setting time, extraction time, extract concentration, cell exposure duration, and the principal cytotoxic effects observed under the different experimental conditions.

Year	Study	Tested Sealers and Controls	Materials Conditions (Setting Time)	Extraction Time	Extract Concentration	Cell Exposure Time	Cytotoxic Potential Effects
2025	Chen Z. et al. [27]	CRoot SP, i-MTA SP, iRoot SP, and NeoSEALER Flo Negative control = medium	Set (24 h)	3 d	0.2 mg/mL, 2 mg/mL, 5 mg/mL, 10 mg/mL, 20 mg/mL, and 50 mg/mL	24 h and 2 d	All sealers demonstrated a dose-dependent response and time-dependent increase in cytotoxicity.
	Gaafar S. S. et al. [28]	CeraSeal and NeoSEALER Flo Positive control = AH Plus	Set (24 h)	24 h	25%, 50%, 75%, and 100% relative to volume	1 d, 3 d, and 7 d	NeoSealer Flo and CeraSeal bioceramic sealers exhibited concentration-dependent cytotoxicity. CeraSeal had significantly higher viability than AH Plus on day 7. NeoSEALER Flo exhibited significant viability differences between days 1 vs. 3 and 1 vs. 7. CeraSeal showed significant changes between days 1 vs. 3, 1 vs. 7, and 3 vs. 7, while AH Plus differed significantly between days 1 vs. 3 and 1 vs. 7.
	Kwan D.C.Y. et al. [29]	AH Plus, AH Plus Bioceramic, CRoot SP, and iRoot SP Negative control = medium	Set (3 d)	3 d	1:2, 1:5, and 1:10	3 and 7 days	On day 3: all sealers exhibited significant toxicity at the 1:1 dilution. AH Plus showed the highest cytotoxicity across all dilutions. At the 1:5 and 1:10 dilutions, CRoot SP, iRoot SP, and AH Plus Bioceramic showed no cytotoxicity. At the 1:10 dilution, CRoot and iRoot displayed significant increase in vitality. On day 7: all sealers remained cytotoxic at the 1:1 dilution. AH Plus was significantly more cytotoxic at the 1:2 dilution, while other sealers exhibited similar viability relative to the control. At the 1:5 dilution, all sealers approached 100% relative cell viability; however, AH Plus remained significantly cytotoxic compared to the other sealers. At the 1:10 dilution, there were no statistically significant differences in cell viability between the sealers and the control.
	Nashibi S. et al. [30]	MTA Angelus and AGM MTA Negative control = medium	Fresh (30 min) Set (24 h)	24 h	100%, 50%, and 25%	24 h and 3 d	MTA Angelus and AGM MTA exhibited concentration-dependent cytotoxicity, indicating that neither diluted extract materials elicited any cytotoxic effects after 24 h or 3 d of exposure. Cements with a 24 h setting time exhibited greater cell viability compared to 30 min set materials. Regarding exposure time, all the materials demonstrated greater cytotoxicity after 3 d of exposure compared to 24 h. Undiluted (100%) extracts of AGM MTA were significantly more cytotoxic than MTA Angelus. For the other concentrations, there were no statistically significant differences.
	Pitzschk M. A. L. R. et al. [31]	AH Plus Jet, Bio-C Sealer, and EndoSequence BC HiFlow Negative control = medium	Set (2 d)	24 h	1:1 and 1:5	24 h, 2 d, and 3 d	AH Plus Jet showed good biocompatibility, like the control, at 24 and 48 h. EndoSequence BC HiFlow and Bio-C Sealer were more cytotoxic, especially in undiluted form. After 72 h, they remained more toxic than AH Plus Jet. However, dilution reduced Bio-C Sealer’s toxicity, making it comparable to AH Plus Jet. Overall, AH Plus Jet was the most stable and biocompatible sealer over time.
	Ramos R.F. et al. [32]	Bio-C Sealer, Bio-C Sealer ion +, and AH Plus Negative control = medium	Set (2 d)	24 h	1:1, 1:2, and 1:4	24 h	All sealer extracts decreased hPDLSC viability in a dose-dependent way compared to the control group. All sealers cause a significant reduction in cell viability at a 1:1 dilution. At a 1:2 dilution, only AH Plus causes a statistically significant reduction in cell viability. At a 1:4 dilution, no material caused a significant reduction in cell viability.
	Santiago M.C. et al. [33]	AH Plus Bioceramic, Bio-C Sealer, NEOMTA Plus, and MTA Fillapex Negative control = medium	Set (3 d)	-	-	24 h	After 24 h of exposure to the AH Plus Bioceramic, the viability of human dental pulp cells (hDPCs) was significantly lower compared to the NEOMTA Plus, Bio-C, and control groups. In contrast, osteoblastic Saos-2 cells exposed to AH Plus Bioceramic and NEOMTA Plus demonstrated viability rates comparable to the control group, while the viability of Saos-2 cells exposed to Bio-C was significantly lower than that of the control group. Additionally, both hDPCs and Saos-2 cells exposed to MTA Fillapex exhibited the lowest viability rates among all groups.
	Wang Z. et al. [34]	iRoot SP and NeoSEALER Flo Negative control = medium	Set (3 d)	3 d	0.2, 2, 10, 20, and 50 mg/mL	24 h and 2 d	Both materials exhibited concentration-dependent effects on cell viability. At 24 h, cells treated with iRoot SP (0.2, 2, 5, 10, and 20 mg/mL) demonstrated significantly higher cell viability compared with the control group (0 mg/mL), whereas NeoSEALER Flo at equivalent concentrations showed no significant effect. Moreover, iRoot SP showed a more favorable effect on cell viability compared with NeoSEALER Flo at 20 mg/mL. At 48 h, tested concentrations (0.2, 2, 5, 10, and 20 mg/mL) of iRoot SP exerted no significant impact on macrophage viability compared with the control group. The viability of macrophages treated with 0.2 mg/mL NeoSEALER Flo was comparable with the control group. In contrast, concentrations of 2, 5, 10, and 20 mg/mL of NeoSEALER Flo significantly promoted cell proliferation compared with the control group.
	Ye Y. et al. [35]	EndoSequence BC Sealer, AH Plus Bioceramic, and AH Plus Jjet Negative control = medium	Fresh (4 h) Set (24)	24 h	1:2, 1:4, 1:8, 1:16, 1:32, and 1:64	1, 3, and 7 days	Cell proliferation rates were significantly influenced by dilution, cell exposure time, and sealer setting conditions. AH Plus Jet showed the lowest proliferation rates across all tested dilutions. EndoSequence BC and AH Plus Bioceramic extracts both demonstrated significantly higher cell proliferation compared to AH Plus Jet, with EndoSequence BC extracts achieving the highest proliferation rates. EndoSequence BC fresh extracts at 1:64 dilution significantly increased cell viability after 3 days relative to the negative control.
2024	Chen J. H. et al. [36]	AH Plus Jet, AH Plus Bioceramic, BioRoot RCS, and BioRoot Flow Negative control = HBSS	Set AH Plus Jet (8 h) Set AH Plus Bioceramic (4 h) Set BioRoot RCS (5.4 h) Set BioRoot Flow (8 h)	24 h, 28 d, and 90 d	1:4	24 h	None of the leachates affected cell viability.
	Çelebi Keskin İ. S. et al. [37]	Sealapex, Apexit Plus, AH Plus, TotalFill BC, and MTA Fillapex Negative control = medium	Set (24 h)	24 h	1:0, 1:1, 1:2, 1:4, and 1:8	24 h and 3 d	At 24 h, TotalFill BC showed significantly higher cell viability than all other sealers across all dilutions. Across all dilutions, AH Plus exhibited the highest cytotoxicity; however, its toxicity was comparable to that of MTA-Fillapex at some dilutions. Sealapex showed significantly higher viability than Apexit Plus at the 1:0 dilution, whereas no significant differences were observed between the two sealers at the other dilutions. MTA-Fillapex showed higher viability than AH Plus at the 1:2 and 1:8 dilutions, while at the remaining dilutions its cell viability was comparable to that of AH Plus. At 3 d, TotalFill BC again showed the highest viability at all dilutions. AH Plus remained the most toxic sealer, except at the 1:8 dilution of Sealapex and MTA-Fillapex. Sealapex, Apexit Plus, and MTA-Fillapex showed similar viabilities; however, Apexit Plus exhibited higher viability than MTA-Fillapex at the 1:0, 1:2, and 1:8 dilutions.
	López-García S. et al. [38]	Neosealer Flo, AH Plus Bioceramic, and TotalFill BC Negative control = medium	Set (2 d)	24 h	1:1, 1:2, and 1:4	24 h, 2 d, and 3 d	Neosealer Flo extracts showed lover cell viability at all dilutions and times evaluated compared to the negative control except for 1/2 and 1/4 dilutions at 24 h. Cells treated with 1/2 and 1/4 dilutions of TotalFill BC Sealer showed a mitochondrial activity like the negative control group. AH Plus Bioceramic-treated cells showed a discrete mitochondrial activity compared to the control group.
	Sanz J. L et al. [39]	BioRoot Flow, AH Plus Bioceramic, and AH Plus Negative control = medium	Set (2 d)	24 h	1:1, 1:2, and 1:4	24 h, 2 d, and 3 d	AH Plus: At all measurement time points and dilutions, AH Plus-treated cells exhibited a significantly lower viability. AH Plus Bioceramic: At 1:2 and 1:4 dilutions, AH Plus Bioceramic-treated cells exhibited non-significant differences with the control group at every measurement time point, while cells treated with undiluted AH Plus bioceramic showed significantly lower viability after 3 days. Bio Root Flow: At 1:4 dilutions, Bio Root Flow-treated cells exhibited non-significant differences with the control group at every measurement time point.
	Zhou G. et al. [40]	CRoot SP and iRoot SP AH Plus Negative control = medium	Set (3 d)	3 d	0.02, 0.2, 2, 5, and 10 mg/mL	24 h, 3 d, and 5 d	At 0.02 and 0.2 mg/mL, no significant differences in cell viability were observed for the three sealers compared with the control group at any time point. At 2 mg/mL, iRoot SP-treated cells showed a statistically significant reduction in cell viability compared with the control group at all measurement time points. At 5 and 10 mg/mL, both iRoot SP- and AH Plus-treated cells showed a statistically significant reduction in cell viability compared with the control group at all time points. In contrast, CRoot SP-treated cells showed a statistically significant reduction in cell viability only after 3 and 5 days of exposure. Cell viability of CRoot SP was consistently higher than that of iRoot SP at both 5 and 10 mg/mL across all time points. The maximum cytotoxic effect was observed on day 5 at 10 mg/mL for AH Plus.
2023	Kandemir Demirci G. et al. [41]	AH Plus Bioceramic, TotalFill BC, AH Plus, and AH Plus Jet Positive control = Doxorubicin	Set (2 d)	Direct contact test: - Indirect contact test: 24 h	Direct contact test: - Indirect contact test: 1:1, 1:2, and 1:4	Direct contact test: 24 h Indirect contact test: 24 h, 2 d, and 3 d	AH Plus Bioceramic and TotalFill BC showed lower cytotoxicity than AH Plus and AH Plus Jet. The cytotoxicity of the sealers increased over time and with higher concentrations. In the indirect contact test, the cell viability values of all tested materials were significantly different between SAOS-2 and THP-1 cells, and between hPDLF and THP-1 cells. The highest cell viability was observed in SAOS-2 cells, followed by hPDLF and THP-1 cells.
	Melo A. P. et al. [42]	BioRoot RCS, Bio-C Sealer, and Sealer Plus Bioceramic Negative control = medium	Set (24 h)	24 h	1:1, 1:2, and 1:5	24 h	At a 1:1 dilution, all tested sealers significantly reduced cell viability compared to the control. At a 1:2 dilution, SealerPlus Bioceramic showed lower cell viability than BioRoot and Bio-C. At a 1:5 dilution, BioRoot and SealerPlus Bioceramic exhibited higher cell viability compared to the control. Overall, increasing the dilution of the eluate reduced cytotoxicity.
	Nguyen L. C. H. et al. [43]	CeraSeal and AH Plus Negative control = medium	Set (2 d)	24 h	1:1, 1:2, 1:4, and 1:8	24 h, 2 d, and 3 d	The cell viability percentages of hSCAPs exposed to undiluted CeraSeal extract were significantly higher than AH Plus over all application times. At 1:4 and 1:8 dilutions for all observed periods, there were no significant differences in cell viability between two kinds of sealer extraction and between CeraSeal and the control. The cell viability of cells treated with CeraSeal extracts at 1:1 and 1:2 dilutions showed a statistically significant reduction after 24 h compared to the negative control. In contrast, at 3 days, the 1:2 dilution of the CeraSeal extract exhibited a significant increase in cell viability compared to the control group.
	Yan Y. et al. [44]	EndoSequence BC HIFlow, iRoot SP, and AH Plus Negative control = medium	Set (2 d)	24 h	1:4 and 1:8	24 h, 3 d, and 7 d	At 7 days, AH Plus showed no significant cytotoxic effect on hSCAPs viability compared to the control. iRoot SP promoted cell proliferation at 1:4 dilution but had no significant effect at 1:8 dilution. EndoSequence BC HiFlow promoted hSCAPs proliferation at both dilutions (1:4 and 1:8), with a stronger effect at 1:4 dilution.
2022	Oliveira P. Y. et al. [45]	Endofill, Sealer 26, White MTA, and Pulp Canal Sealer Negative control = sterilized polyethylene capillary tubes	Set (24 h)	-	-	24 h and 2 d	At 24 h, no material was cytotoxic. At 2 days, only Pulp Canal Sealer and Endofill showed a statistically significant reduction in viability; MTA and Sealer 26 did not affect cell viability under any of the conditions tested.
	Pedrosa M. D. S. et al. [46]	Bio-C Sealer, MTA Fillapex, and Cimmo HP Negative control = medium	Set (24 h)	3 d	1:1, 1:4, and 1:16	24 h, 2 d, and 3 d	Bio-C Sealer was cytotoxic in its undiluted form (1:1) at 24 h to LPS-activated cells, and at 2 and 3 days for both LPS-activated and untreated cells. However, diluted forms (1:4 and 1:16) increased cell viability, especially in LPS-activated cells at 2 and 3 days. LPS-activated cells generally showed higher viability than untreated ones. Cimmo HP reduced cell viability in its undiluted form at all time points. At 2 days, only the 1:4 and 1:16 dilutions were non-cytotoxic to LPS-activated cells. MTA Fillapex in undiluted form showed cytotoxicity across all time points in both untreated and LPS-activated cells.
	Rosatto C. M. P. et al. [47]	EndoSequence BC, Bio-C Sealer, and Sealer Plus Bioceramic Negative control = medium	Set (24 h)	24 h	1:20, 1:100, 1:500, and 1:2500	12 h, 24 h, and 2 d	A significant reduction in cell viability was observed for all extract dilutions at all time points for EndoSequence BC, Bio-C, and Sealer Plus Bioceramic sealers, compared to the control. EndoSequence BC showed similar cell viability across various extract dilutions and time points, except at dilutions of 1:20 to 1:2500 after 2 days, where a reduction was observed. The Bio-C and Sealer Plus groups showed a significant reduction in the percentage of cell viability at 2 days for all dilutions.
	Sanz J. L. et al. [48]	AH Plus Bioceramic, AH Plus, and EndoSequence BC Negative control = medium	Set (2 d)	24 h	1:1, 1:2, and 1:4	24 h, 2 d, and 3 d	Cells treated with EndoSequence BC and AH Plus Bioceramic showed similar viability to that of the control group at all time points and concentrations analyzed, except at the 1:1 concentration, where cells treated with AH Plus Bioceramic exhibited significantly lower viability after 72 h of exposure compared to the control group. Cells treated with AH Plus showed significantly lower viability compared to the control group at all time points and concentrations.
	Tomokiyo A. et al. [49]	SuperBond sealer, Nishika Canal Sealer BG, and GuttaFlow 2 Negative control = medium	Set (24 h)	-	-	3 d	Cell counting: The number of cells in the GuttaFlow 2 group was comparable to that of the control group. In contrast, cell counts were significantly lower in the SuperBond Sealer and Nishika Canal Sealer BG groups than in the control. MTT assay: All tested groups showed reduced cell viability compared to the control group. Among them, the SuperBond Sealer group exhibited the lowest cell viability, followed by Nishika Canal Sealer BG, and then GuttaFlow 2.
	Wuersching S. N. et al. [50]	AH Plus, GuttaFlow bioseal, BioRoot RCS, and TotalFill BC Negative control = medium	Set (24 h)	24 h and 7 d	Undiluted, 1:1, and 1:5	24 h	hPDLF, 24 h sealer eluates: Cytotoxicity was observed with undiluted eluates of AH Plus, GuttaFlow Bioseal, and BioRoot RCS. At 1:1 and 1:5 dilutions, cytotoxicity persisted for AH Plus and BioRoot RCS. No cytotoxicity was observed for TotalFill BC eluates. hPDLF, 7-day sealer eluates: Cytotoxicity was observed with undiluted and 1:1 diluted eluates of AH Plus and BioRoot RCS. At the 1:5 dilution, no sample showed a significant difference from the control. TotalFill BC eluates showed no cytotoxicity. hOB, 24 h: Cytotoxicity was observed with undiluted and 1:1 diluted eluates for all tested materials. At the 1:5 dilution, AH Plus, GuttaFlow Bioseal, and TotalFill BC still showed reduced cell viability compared to the control. hOB, 7-day: AH Plus showed a significant reduction in cell viability under all dilution conditions. TotalFill BC showed a significant reduction in the undiluted condition only. GuttaFlow Bioseal and BioRoot RCS did not show a significant reduction in viability compared to the control. Increased dilution resulted in reduced cytotoxicity of the eluates.
2021	Dhopavkar V. V. et al. [51]	AH Plus, MTA Filapex, and GuttaFlow2 Negative control = medium	Set (24 h)	-	-	24 h and 2 d	AH Plus and GuttaFlow 2 did not exhibit any cytotoxicity throughout the tested time intervals (24 h and 2 d). MTA Fillapex showed higher cytotoxicity compared to AH Plus and GuttaFlow 2.
	Erdogan H. et al. [52]	AHPlus, MTA-Fillapex, and iRoot SP Negative control = medium	Fresh Set (6 h, 12 h, 24 h, 2 d, and 3 d)	24 h	1:2, 1:4, 1:8, 1:16, and 1:32	24 h	AH Plus: The maximum cytotoxic effect was observed at a concentration of 1:1 in the fresh state. The maximum cytotoxic effect was observed at a 1:1 concentration, and a significant difference was seen between high concentrations (1:1, 1:2, and 1:4) and the control group at 6 and 12 h of setting time. MTA Fillapex: Low viability rates were observed at high concentrations (1:1, 1:2, and 1:4), and there was a difference between these concentrations and the control group in the fresh state and after the 6 h of setting time. iRootSP: Viability percentages were high at all time points. There was no statistically significant difference between any concentrations and the control group. Comparative evaluation of cytotoxic effects of different root canal sealers: In the fresh state, the statistical relationship between the cytotoxic effects of the sealers at the 1:1 concentration was determined as follows: AH Plus = MTA Fillapex > iRoot SP. A statistically significant difference was observed between AH Plus and iRoot SP at concentrations of 1:2 and 1:4. After 6 h of setting time, the highest cytotoxic effect at the 1:1 concentration was observed for AH Plus. The statistical relationship was AH Plus = MTA Fillapex > iRoot SP. At 1:2 and 1:4 concentrations, a significant difference was observed between AH Plus and iRoot SP. At 12 and 24 h, a statistically significant difference was observed between AH Plus and iRoot SP at the 1:1, 1:2, and 1:4 concentrations, with iRoot SP showing the least cytotoxic effect.
	Kan M. T. et al. [53]	AH Plus, GuttaFlow 2, Dia-ProSeal, and Pulpdent Root Canal Sealer Negative control = medium	Fresh Set (24 h)	24 h	Undiluted	24 h, 3 d, and 7 d	Fresh samples: The Dia-ProSeal samples showed a clear trend of increasing cell viability over time. In contrast, no consistent trend in survival rates was observed across days 1, 3, and 7 for the AH Plus, GuttaFlow 2, and Pulpdent Root Canal Sealer groups. For AH Plus, GuttaFlow 2, and Pulpdent Root Canal Sealer, the highest cell viability was recorded on day 3. The cell viability of freshly mixed AH Plus, Dia-ProSeal, and Pulpdent Root Canal Sealer samples on day 1 was significantly lower compared to days 3 and 7. However, the difference in cell viability between days 3 and 7 for these three groups (AH Plus, Dia-ProSeal, and Pulpdent Root Canal Sealer) was not statistically significant. For GuttaFlow 2, there was no significant difference in cell viability between days 1 and 7. However, a significant increase in cell viability was observed on day 3 compared to both days 1 and 7. Set samples: The difference in cell viability between all days was significant for AH Plus and Dia-ProSeal samples. For GuttaFlow 2 and Pulpdent Root Canal Sealer samples, there was a significant reduction on days 3 and 7 compared to day 1. A clear trend was observed for all set groups, with a decrease in cell viability as the days progressed. All samples showed the maximum cell viability of fibroblast cells on day 1 and a minimum cell viability on day 7. Comparison of fresh and set samples: The comparison of the cell viability of all freshly mixed sealers and their hardened forms revealed that the difference was statistically significant on day 1, with an increase in viability in the hardened form. The comparison of the cell viability of freshly mixed AH Plus, Dia-Proseal, and Pulpdent root canal sealants and their hardened forms revealed a statistically significant difference on day 3. For GuttaFlow 2, the difference in cell viability between the freshly mixed and hardened forms was not statistically significant on day 3. On day 7, a comparison of the cell viability of all freshly mixed sealants and their hardened forms revealed that the difference was statistically significant.
	Park M. G. et al. [54]	AH Plus, BrightEndo MTA, CeraSeal, EndoSeal TCS, and One-Fil Negative control = medium	Fresh Set (2 d)	Fresh: 24 h Set: 2 d and 3 d	1/4	Fresh: 24 h, 2 d, 3 d, and 7 d Set: 24 h, 3 d, and 7 d	Fresh samples: AH Plus showed significantly lower cell viability than all other sealers at every time point. BrightEndo MTA exhibited significantly lower cell viability than the control group on day 3, while on day 7 CeraSeal and EndoSeal TCS showed significantly higher cell viability compared to the control. BrightEndo MTA, CeraSeal, EndoSeal TCS, and One-Fil demonstrated a consistent increase in cell viability over time. Set samples: 2-day extraction time: All sealers showed an increasing trend in cell viability over time. No statistically significant differences from the control group were observed until day 3. However, on day 7, AH Plus and One-Fil exhibited significantly higher cell viability compared to the control. 3-day extraction time: AH Plus showed significantly higher cell viability than the control on day 1, but it was significantly lower on day 3. One-Fil demonstrated significantly lower cell viability on day 3 and significantly higher cell viability on day 7 compared to the control. BrightEndo MTA, CeraSeal, and EndoSeal TCS showed no significant differences from the control at any time point.
	Pawinska M. et al. [55]	Endomethasone N, Roeko Seal Automix, RealSeal, and Sealapex Negative control = medium	Fresh Set (24 h)	-	-	24 h	Fresh samples: All sealers caused a significant reduction in cell viability after 24 h exposure compared to the control group. Sealapex, RealSeal, and Roeko Seal Automix showed the lowest percentages of viable cells, with no significant differences between them. Endomethasone N showed the highest percentage of viable cells and was significantly less cytotoxic than Sealapex and RealSeal. RealSeal and Roeko Seal Automix significantly increased apoptosis, while Sealapex and Endomethasone N significantly increased necrosis compared to the other sealers and the control group. Set samples: All set materials caused a significant reduction in cell viability after 24 h incubation compared to the control group. The lowest viability was observed for RealSeal, followed by Endomethasone N and Sealapex, with no significant differences among them. Roeko Seal Automix showed the highest cell viability, comparable to the control, and was significantly less cytotoxic than RealSeal and Endomethasone N. RealSeal significantly increased both apoptosis and necrosis, while Sealapex, Endomethasone N, and Roeko Seal Automix significantly increased necrosis compared to the control group. Comparison of fresh and set samples: Fresh materials induced a lower percentage of viable cells compared to set materials for RealSeal, Sealapex, and Roeko Seal Automix, though the differences were not statistically significant. Endomethasone N showed an opposite trend, with slightly higher viability in the fresh condition, but again without statistical significance. RealSeal, Sealapex, and Roeko Seal Automix significantly increased apoptosis both immediately after mixing and after setting. For Endomethasone N, a non-significant increase in necrosis was observed with fresh materials. Set RealSeal and Roeko Seal Automix induced significantly more necrosis than their fresh counterparts.
	Pedrosa M. D. S. et al. [56]	Bio-C Sealer, MTA Fillapex, and Cimmo HP Negative control = medium	Set (24 h)	3 d	1, 1:2, 1:4, 1:8, and 1:16	24 h, 2 d, and 3 d	At 24 h, Bio-C Sealer at a 1:8 dilution showed significant cytotoxicity compared to the negative control, while no significant differences were observed for any dilution of Cimmo HP and MTA Fillapex when compared to the negative control. At 2 days, Bio-C Sealer showed no cytotoxicity at any dilution. Undiluted Cimmo HP was statistically significantly cytotoxic compared to the negative control, but its 1:16 dilution showed improved viability. MTA Fillapex at the 1 and 1:2 dilutions was cytotoxic. At 3 days, undiluted Bio-C Sealer and Cimmo HP, as well as MTA Fillapex at 1, 1:2, and 1:4 dilutions, were cytotoxic. Bio-C Sealer at a 1:16 dilution showed a significantly higher absorbance than the control and other dilutions.
	Zordan-Bronzel C. L. et al. [57]	Sealer Plus Bioceramic, TotalFill BC, and AH Plus Negative control = medium Positive control = 20% dimethyl sulfoxide	Set (24 h)	24 h	1:1, 1:2, 1:4, 1:8, 1:16, and 1:32	MTT assay: 1:1, 1:2, 1:4, 1:16, and 1:32 = 24 h 1:8 = 24 h, 3 d, and 7 d Neutral Red assay: 24 h	In the MTT assay, only Sealer Plus Bioceramic showed significantly lower cell viability at the 1:1 and 1:2 dilutions compared to all other sealers and the negative control. At the 1:4 dilution, Sealer Plus Bioceramic did not show any statistically significant differences from the control or the other materials. At higher dilutions, this sealer resulted in a statistically significant increase in cell viability compared to the control and the other materials analyzed. In the Neutral Red assay, AH Plus, Sealer Plus Bioceramic, and TotalFill BC showed no cytotoxicity, with cell viability not significantly different from the negative control. At 1 and 7 days, Sealer Plus Bioceramic showed significantly higher cell viability compared to the control group.
2020	Chen B. et al. [58]	EndoSequence BC HiFlow and EndoSequence BC Negative control = medium	Set (2 d)	24 h	1:4, 1:8, 1:16, and 1:20	24 h, 2 d, and 3 d	No significant differences in cell viability were observed between EndoSequence BC and Endosequence BC HiFlow. For both sealers, fibroblasts exposed to 1:4 diluted extract for 3 days showed significantly reduced viability compared to other incubation times and extract concentrations. At dilutions of 1:8 or higher, no cytotoxic effects were observed at any incubation time.
	López-García S. et al. [59]	Endoseal MTA, Ceraseal, and EndoSequence BC Negative control = medium	Set (2 d)	24 h	1:1; 1:2, and 1:4	24 h, 2 d, and 3 d	Ceraseal and EndoSequence BC showed cell viability similar to that of the control. At 3 d, undiluted Ceraseal and EndoSequence BC at a 1:2 dilution resulted in a significant increase in viability. In contrast, Endoseal at all dilutions significantly reduced cell viability at 24 h, 2 d and 3 d.
	Rodríguez-Lozano F. J. et al. [60]	EndoSequence BC HiFlow, EndoSequence BC, and AH Plus Negative control = medium	Set (2 d)	24 h	Undiluted, 1:2, and 1:4	24 h, 2 d, and 3 d	At 24 h, undiluted EndoSequence BC HiFlow and EndoSequence BC significantly increased cell viability compared to the control, while AH Plus significantly reduced it. At 2 d and 3 d, EndoSequence BC HiFlow and EndoSequence BC showed no significant differences from the control, whereas AH Plus continued to significantly reduce cell proliferation. At a 1:2 dilution, EndoSequence BC HiFlow and EndoSequence BC increased viability at 24 h, but not at 2 d or 3 d. AH Plus showed a consistent reduction in viability at all time points and dilutions. At a 1:4 dilution, EndoSequence BC HiFlow and EndoSequence BC showed no cytotoxic effect, while AH Plus maintained the same cytotoxicity as at higher concentrations.
2019	Jeanneau C. et al. [61]	BioRoot RCS and Pulp Canal Sealer Negative control = medium	Fresh	24 h	0.2 mg/mL	3 d, 6 d, and 9 d	BioRoot RCS extracts increased cell proliferation at 6 and 9 days, while Pulp Canal Sealer extracts significantly reduced proliferation at the same time points.
	Jung S. et al. [62]	AH Plus, MTA Fillapex, BioRoot RCS, and Pulp Canal Sealer Negative control = medium	Fresh Set (2 d)	24 h	1:1, 1:2, and 1:10	24 h, 7 d, 14 d, and 21 d	In the 1:1 dilution, all cells died regardless of sealer type. At a 1:2 dilution, MTA Fillapex and Pulp Canal Sealer showed significantly reduced cell survival and proliferation compared to controls, with detectable LDH release through day 7. At a 1:10 dilution, these two sealers reached control-level cell viability by day 21. AH Plus was cytotoxic even at 1:10 dilution, causing complete cell death and morphological changes, with high LDH release. In contrast, BioRoot RCS showed no cytotoxicity at 1:2, supported by cell count, MTT, LDH, and staining data, and even promoted cell proliferation.
	Lee B. N. et al. [63]	AH Plus, MTA Fillapex, and EndoSequence BC Negative control = medium	Set (24 h)	7 d	1, 1:5, 1:10, 1:50, and 1:100	1 d	AH Plus significantly reduced cell viability at all dilutions. MTA Fillapex and EndoSequence BC Sealer showed significant cytotoxicity only at high concentrations.
	Lee J. K. et al. [64]	AH Plus, AD Seal, EndoSeal MTA, Nano-Ceramic Sealer, and Wellroot ST Negative control = n/s	Fresh Set (2 d)	Fresh: 24 h Set: 2 d and 3 d	1/4	Fresh: 24 h, 2 d, 3 d, and 7 d Set: 24 h, 3 d, and 7 d	In the fresh state, the cell viability of AH Plus, Wellroot ST, and EndoSeal MTA decreased over time. In 2-day set extracts, no significant differences were observed among materials. In 3-day set extracts, Wellroot ST showed an increase in cell viability on day 3 and Nano-ceramic Sealer significantly increased viability over the 7-day period. AH Plus showed the lowest cell viability throughout the 7-day experiment.
	López-García S. et al. [65]	Bio-C Sealer, TotalFill BC, and AH Plus Negative control = medium	Set (2 d)	24 h	Undiluted, 1/2, and1/4	24 h, 2 d, and 3 d	The metabolic activity of cells varied according to time and material. At 24 h, TotalFill BC and Bio-C Sealer did not reduce cell viability, while AH Plus showed significant cytotoxicity at all dilutions. At 2 d, all materials at 1:2 dilution caused significant reductions in cell viability compared to the control. At 3 d, TotalFill BC Sealer at 1:2 and 1:4 dilutions led to a significant increase in cell viability compared to the negative control, while Bio-C Sealer at 1:4 showed no significant difference from the control. Overall, TotalFill BC Sealer and Bio-C Sealer were significantly less cytotoxic than AH Plus at all dilutions.
	Rodríguez-Lozano F. J. et al. [66]	AH Plus, MTA Fillapex, GuttaFlow 2, and GuttaFlow Bioseal Negative control = plastic	Set (2 d)	24 h	Undiluted	3 d	After 72 h of incubation, undiluted extracts of GuttaFlow Bioseal and GuttaFlow2 maintained high viability rates of cells, comparable to the control group. In contrast, AH Plus and MTA Fillapex showed significant cytotoxic effects.
	Seo D. G. et al. [67]	AH Plus, EndoSequence BC, BioRoot RCS, and Endoseal MTA Negative control = hDPSCs cultured without experimental disks	Set (3 d)	-	-	24 h, 2 d, 3 d, and 5 d	After 72 h, AH Plus showed the lowest cell viability among all groups. No significant differences in cell viability were observed between EndoSequence BC, BioRoot RCS, Endoseal MTA, and the control group at any experimental time point.
2018	Alsubait S. A. et al. [68]	BioRoot RCS, Endosequence BC, and AH Plus Jet Negative control = medium	Fresh	24 h	1:2, 1:8, and 1:32	24 h, 3 d, and 7 d	At each time point, the number of cells exposed to AH Plus Jet was significantly lower than in the control group, except at the 1:32 dilution at 24 h. No significant difference was detected in cell viability between the Endosequence BC and the control at any time point. BioRoot RCS at 1:2 dilution showed a significant statistical reduction in cell viability at each time point, while no significant difference in cell viability was detected between the 1:8 or 1:32 dilutions for BioRoot RCS or the control at any time point.
	Jung S. et al. [69]	AH Plus, Pulp Canal Sealer, MTA Fillapex, and BioRoot RCS Negative control = medium	Set (2 d)	24 h	1:1, 1:2, and 1:10	24 h, 7 d, 14 d, and 21 d	At the undiluted concentration, all cells died within the first 24 h, regardless of sealer type. At a 1:2 dilution, AH Plus and Pulp Canal Sealer induced complete cell death within the first few days. MTA Fillapex at the same dilution allowed osteoblast survival and metabolic activity up to day 7, as evidenced by the MTT assay, with LDH release continuing until day 14. However, live/dead staining showed almost no viable cells after 14 and 21 days, indicating progressive cytotoxic effects during this period. In contrast, BioRoot RCS at a 1:2 dilution supported osteoblast survival and proliferation, with significantly higher metabolic activity compared to the control, except on day 1. At a 1:10 dilution, osteoblasts did not survive exposure to AH Plus extract, in contrast to all other sealers tested. Live/dead staining confirmed a very low cell density after 14 and 21 days, with altered cell morphology, showing enlarged cells with prolonged incubation. For all sealers tested at a 1:10 dilution, a progressive increase in LDH release was observed over time, indicating ongoing cytotoxic effects at this dilution.
	Martinho F. C. et al. [70]	Real Seal, AH Plus, EndoRez, and Apexit Negative control = medium	Set (24 h)	24 h	1:1, 1:2, 1:4, 1:8, and 1:16	24 h	At a 1:2 dilution, all root canal sealers led to reduced cell viability, with no statistically significant difference compared to the control group, except for Real Seal, which showed significantly higher cytotoxicity compared to the control group. At the highest dilution tested (1:16), none of the sealers exhibited cytotoxic effects. Intragroup analysis confirmed that Real Seal had the highest cytotoxicity overall. No significant differences in cytotoxicity were found among AH Plus Jet, EndoREZ, and Apexit Plus.
	Szczurko G. et al. [71]	AH Plus Jet, Apexit Plus, MTA Fillapex, GuttaFlow, MetaSEAL Soft, and Tubli-Seal Negative control = medium	Fresh Set (24 h)	-	-	24 h	In the fresh state, all materials reduced periodontal fibroblast viability compared to the control. The order of cytotoxicity was MetaSEAL Soft > AH Plus > Tubli-Seal > MTA Fillapex > Apexit Plus > GuttaFlow. Fresh MetaSEAL Soft was significantly more cytotoxic than all other materials. AH Plus and Tubli-Seal also showed significantly higher toxicity compared to MTA Fillapex, Apexit Plus, and GuttaFlow. Fresh GuttaFlow and Apexit Plus did not exhibit cytotoxicity and even promoted cell proliferation. In the set state, the same overall ranking of cytotoxicity was observed: MetaSEAL Soft > AH Plus > Tubli-Seal > MTA Fillapex > Apexit Plus > GuttaFlow. Set MetaSEAL Soft remained the most toxic material. Set AH Plus and Tubli-Seal were still more cytotoxic than MTA Fillapex, Apexit Plus, and GuttaFlow, and set MTA Fillapex was significantly more toxic than set Apexit Plus and GuttaFlow. Set GuttaFlow and Apexit Plus maintained their non-cytotoxic behavior and continued to stimulate fibroblast proliferation. Additionally, AH Plus, Tubli-Seal, and MTA Fillapex were significantly less toxic in their set forms than in their fresh counterparts.
	Taraslia V. et al. [72]	MTA Fillapex, GuttaFlow 2, EndoSequence BC, Bioroot RCS, AH Plus, and Roth’s 801 Negative control = Transwell system without material	Fresh Set (24 h)	-	-	3 d	GuttaFlow 2, EndoSequence BC, MTA Fillapex, and BioRoot demonstrated significantly higher cell viability than Roth’s 801 and AH Plus, except for MTA Fillapex and BioRoot in the freshly mixed state, where no significant differences were detected. In both freshly mixed and set conditions, GuttaFlow 2 showed the highest number of viable cells. Significant differences in cell viability between the fresh and set states were observed for Roth’s 801, GuttaFlow 2, and EndoSequence BC, with the set forms showing improved biocompatibility. MTA Fillapex and BioRoot also showed higher viability in their set forms compared to the freshly mixed state, but these differences were not statistically significant.
	Vouzara T. et al. [73]	SimpliSeal, MTA Fillapex, and BioRoot RCS Negative control = medium	Set (2 d)	24 h and 7 d	1:1 and 1:2	24 h and 3 d	BioRoot RCS was significantly less cytotoxic than both MTA Fillapex and SimpliSeal at all concentrations and time points. Cytotoxicity increased over time, with extracts obtained after one week being more toxic than those after 24 h. MTA Fillapex and SimpliSeal showed comparable antiproliferative effects, with no significant differences observed between them.
2017	Arun S. et al. [74]	Tubliseal, AH Plus, Sealapex, and EndoREZ Negative control = medium	Fresh	-	-	24 h	All four tested sealers exhibited cytotoxicity to L929 cells. Sealapex showed the lowest toxicity, followed by AH Plus, Tubli-Seal, and finally EndoREZ.
	Cintra L.T.A. et al. [75]	Sealer Plus, AH Plus, Endofill, and SimpliSeal Negative control = medium	Set (6 h)	3 d	1:1, 1:2, and 1:4	6 h, 24 h, 2 d, and 3 d	At the 6 h time point, undiluted Sealer Plus exhibited the highest cell viability among all undiluted sealers tested. By 24 h, all dilutions of Sealer Plus demonstrated significantly higher cell viability than both the control group and the other sealers. At this stage, SimpliSeal also showed better biocompatibility compared to AH Plus and Endofill. After 48 h, Sealer Plus, both in its undiluted form and at a 1:2 dilution, remained the least cytotoxic. Notably, its 1:2 and 1:4 dilutions were significantly less toxic than the undiluted extract. AH Plus and Endofill also exhibited reduced cytotoxicity at their 1:2 and 1:4 dilutions relative to their undiluted forms. At 72 h, undiluted and 1:2 dilutions of Sealer Plus, along with a 1:4 dilution of Endofill, were the least cytotoxic. SimpliSeal continued to promote higher cell viability than the undiluted forms of AH Plus and Endofill. Nonetheless, all sealers resulted in reduced cell viability when compared to the control.
	Collado-González M. et al. [76]	GuttaFlow Bioseal, Guttaflow 2, MTA Fillapex, and AH Plus Negative control = medium	Set (2 d)	24 h	1:1, 1:2, and 1:4	24 h, 2 d, 3 d, and 7 d	GuttaFlow Bioseal consistently exhibited high biocompatibility. At the 7-day mark, it significantly enhanced cell viability across all tested concentrations, outperforming not only GuttaFlow2, AH Plus, and MTA Fillapex but also the control group. GuttaFlow2 demonstrated similar biocompatibility to GuttaFlow Bioseal at all earlier time points; however, this equivalence was not maintained at 7 days. In contrast, MTA Fillapex significantly reduced cell viability at every time point compared to the control, indicating persistent cytotoxicity.
	Collado-González M. et al. [77]	BioRoot RCS, Endoseal MTA, and Nano-Ceramic Sealer Negative control = medium	Set (2 d)	24 h	1:1, 1:2, and 1:4	24 h, 2 d, and 3 d	BioRoot RCS demonstrated a more biocompatible profile. At the 3-day time point, BioRoot RCS at all tested concentrations significantly increased cell proliferation compared to both Endoseal MTA and Nano-Ceramic Sealer. In contrast, Endoseal MTA significantly reduced cell proliferation compared to the control group at every time point, indicating sustained cytotoxicity.
	Lv F. et al. [78]	iRoot FS, iRoot BP Plus, and ProRoot MTA Negative control = medium	Set (7 d)	3 d	Undiluted, 1:2, and 1:4	24 h, 2 d, and 3 d	The CCK-8 assay results demonstrated that undiluted extracts of all three materials (iRoot FS, iRoot BP Plus, and ProRoot MTA) significantly promoted cell proliferation at all time points compared to the control, with no significant differences observed among the materials. Furthermore, the undiluted extracts induced higher cell viability than their diluted counterparts, indicating a concentration-dependent effect.
	Rodríguez-Lozano F.J. et al. [79]	MTA Fillapex, TotalFill BC, and AH Plus Negative control = medium	Set (2 d)	24 h	1:1, 1:2, and 1:4	24 h, 2 d, and 3 d	MTA Fillapex significantly impaired cell proliferation at all time points and across all dilutions tested. In contrast, both TotalFill BC and AH Plus supported higher levels of proliferation compared to MTA Fillapex. TotalFill BC demonstrated the most favorable biocompatibility profile, with proliferation levels comparable to those of the control group and significantly higher than those of AH Plus from the 48 h time point onward.
2016	Silva E. J. et al. [80]	AH plus, Endomethasone N, EndoSequence BC, MTA Fillapex, and Pulp Canal Sealer EWT Negative control = medium	Fresh	24 h	Undiluted	24 h	Cytotoxicity was significantly higher in the 2D cell culture model than in the 3D model. EndoSequence BC showed the lowest cytotoxicity in both models, whereas MTA Fillapex exhibited the highest. The exception to this was in the 2D model, where MTA Fillapex did not differ significantly from AH Plus. Endomethasone N and Pulp Canal Sealer EWT were less cytotoxic than AH Plus in the 2D model; however, no significant differences were observed among these three sealers in the 3D model.
	Silva E. J. et al. [81]	AH Plus and MTA Fillapex Negative control = medium	Fresh Set (7 d, 14 d, 21 d, and 28 d)	24 h	Undiluted	24 h	Both sealers exhibited strong and comparable cytotoxicity during the first 24 h post-mixing. After one week, AH Plus was no longer cytotoxic in any of the tested parameters. In contrast, MTA Fillapex remained cytotoxic to varying degrees throughout the four-week observation period. Consequently, MTA Fillapex demonstrated significantly higher overall toxicity than AH Plus, except at the initial 24 h time point where no significant difference was observed.
	Suciu I. et al. [82]	MTA Fillapex, AH Plus, and Acroseal Negative control = plastic surface	Set (24 h)	-	-	2 d, 5 d, 9 d, and 14 d	All tested materials reduced cell viability compared to the controls, with the degree of reduction varying by material and cell type. Only AH Plus exhibited significant toxicity toward osteoblasts within 48 h, an effect that persisted up to day 5. From day 9 onward, osteoblast viability remained lower with all materials relative to the control group. In contrast, Acroseal demonstrated higher biocompatibility with DF-MSCs. For this cell type, Acroseal showed viability comparable to the control during the first 5 days and promoted increased proliferation at later time points. Conversely, MTA exhibited the greatest toxicity toward DF-MSCs.
2015	Dimitrova-Nakov S. et al. [83]	BioRoot RCS and Pulp Canal Sealer Negative control = untreated cells	Set (24 h)	-	-	3 d, 7 d, and 10 d	BioRoot RCS showed no statistically significant difference in cytotoxicity compared to the control group. In contrast, Pulp Canal Sealer exhibited significant cytotoxic effects.
	Mestieri L.B. et al. [84]	MTA Plus, MTA Fillapex, and FillCanal Negative control = medium	Set (24 h)	24 h	1:2, 1:3, and 1:4	24 h	MTA Plus exhibited cell viability comparable to the negative control at all tested concentrations. Conversely, both MTA Fillapex and FillCanal demonstrated significantly lower viability than the control across all concentrations. However, at the 1:4 dilution, their viability rates reached levels comparable to those of the MTA Plus group. In the Neutral Red assay, MTA Plus showed a significantly higher viability rate than the control at all dilutions. Among the materials, MTA Fillapex displayed the lowest viability at the 1:2 dilution, but this increased at the 1:3 and 1:4 dilutions. FillCanal maintained good viability at all dilutions and was comparable to MTA Fillapex specifically at the 1:4 dilution.
2014	Camargo C.H. et al. [85]	AH Plus, RoekoSeal, and EndoRez Negative control = medium	Fresh Set (12 h and 24 h)	24 h	1:1, 1:2, 1:4, 1:8, 1:16, and 1:32	24 h	RoekoSeal exhibited nearly 100% cell viability at all tested setting times, indicating minimal cytotoxicity. In contrast, EndoRez was highly cytotoxic at 1:1 and 1:2 dilutions, regardless of the setting period. For AH Plus, cytotoxicity was high at low dilutions but decreased significantly after 12 h of setting time, resulting in low cytotoxicity at 1:2 and 1:4 dilutions. At a 1:8 dilution, AH Plus was cytotoxic only in its freshly mixed state. Notably, none of the sealers exhibited cytotoxicity at the 1:16 and 1:32 dilutions. Overall, the sealers could be ranked by cytotoxicity as follows: EndoRez > AH Plus > RoekoSeal.
	Chang S.W. et al. [86]	Sealapex, Apatite root sealer, MTA Fillapex, and iRoot SP Negative control = medium with or without osteogenic supplements	Set (24 h)	-	-	3 d, 7 d, and 14 d	None of the tested sealers exhibited cytotoxic effects at any of the exposure time points. Among them, MTA Fillapex demonstrated superior cell growth compared to Sealapex, Apatite Root Sealer, and iRoot SP by day 14.
	Cotti E. et al. [87]	RealSeal XT, AH Plus Jet Negative control = untreated cells	Fresh	-	-	1 h, 24 h, 2 d, and 3 d	The Neutral Red assay revealed a time-dependent reduction in cell viability with AH Plus Jet, whereas RealSeal XT maintained viability levels comparable to the control, indicating no significant cytotoxicity. Although AH Plus Jet initially increased viability at the 1 h time point, a marked decline was observed from 24 to 72 h. Similarly, in the MTT assay, AH Plus Jet consistently reduced viability at all time points. In contrast, RealSeal XT preserved higher cell viability throughout the experimental period. Statistical analysis confirmed significant cytotoxic differences over time, which were particularly pronounced between 24 and 72 h. AH Plus Jet exhibited sustained toxicity from 24 h onward. Conversely, RealSeal XT showed only a transient decrease in viability at 24 h, followed by recovery at 48 and 72 h. In summary, AH Plus Jet demonstrated greater cytotoxicity than RealSeal XT in confluent cell cultures.
	Manda P. et al. [88]	AH Plus, ProRoot MTA, RealSeal, and GuttaFlow 2 Negative control = medium	Fresh Set (3 d)	24 h and 3 d	0.5, 1.0, and 1.5 cm^2^/mL	24 h	Set samples: GuttaFlow2, at 0.5 cm^2^/mL, did not significantly affect cell viability at the 24 h extraction time, but the higher concentrations (1.0 and 1.5 cm^2^/mL) caused a significant reduction. By the 3-day extraction time, GuttaFlow2 significantly decreased cell viability at all tested concentrations. AH Plus and RealSeal demonstrated consistent and significant cytotoxicity at all extract concentrations and time points, showing a marked reduction in cell viability compared to the control. For MTA, the lowest concentration (0.5 cm^2^/mL) did not significantly affect cell viability, while the highest concentration (1.5 cm^2^/mL) led to a significant decrease at both the 24 h and 3-day extractions. At 1.0 cm^2^/mL, MTA was cytotoxic only at the 24 h extraction time, with no significant effect observed at 3 days. Fresh samples: In their unset (fresh) state, GuttaFlow2, AH Plus, and RealSeal all significantly reduced cell viability at every concentration and at both extraction time points, indicating a more pronounced cytotoxic effect compared to their set counterparts. Fresh MTA, on the other hand, showed significant cytotoxicity at the 0.5 and 1.0 cm^2^/mL concentrations, while the highest concentration (1.5 cm^2^/mL) did not significantly reduce cell viability at either time point, suggesting a complex dose–response relationship. Overall comparison: Overall, when comparing both fresh and set conditions across all concentrations and time points, GuttaFlow2 and MTA consistently resulted in higher cell viability than AH Plus and RealSeal, indicating a more favorable biological profile.
2013	Guven E.P. et al. [89]	iRoot SP, MTA Fillapex, and AH Plus Jet Negative control = medium	Set (24 h)	-	-	24 h, 3 d, 7 d, and 14 d	MTA Fillapex exhibited significant cytotoxicity compared to the negative control from day 1 onward. However, on day 1, its reduction in cell viability was not significantly different from that caused by the other test groups (iRoot SP and AH Plus Jet). In contrast, on days 3, 7, and 14, MTA Fillapex demonstrated significantly greater cytotoxicity than both iRoot SP and AH Plus Jet. The effects of iRoot SP and AH Plus Jet on hTGSCs also evolved differently. For iRoot SP, a significant reduction in cell viability compared to the negative control was observed only by day 7. AH Plus Jet, however, exhibited a distinct cytotoxic profile that was already significantly different from other groups by day 3.
	Kim T.G. et al. [90]	AH Plus Negative control = medium	Fresh	24 h	30%	24 h	Cells exposed to AH Plus showed a reduction in viability.
2012	Bin C.V. et al. [91]	White MTA, MTA Fillapex, and AH Plus Negative control = medium	Fresh Set (12 h, 2 d and 3 d)	24 h	1:1, 1:2, 1:4, 1:8, 1:16, and 1:32	24 h	White MTA consistently exhibited the lowest cytotoxicity, maintaining high cell viability across all dilutions and time points. Notably, it even promoted cell proliferation, particularly within the first 48 h post-setting, indicating favorable biocompatibility during its early curing phase. In contrast, MTA Fillapex was the most cytotoxic material. Its toxicity was most pronounced at higher concentrations and shortly after manipulation (approximately 12 h), although it decreased at later time points. AH Plus demonstrated moderate cytotoxicity, with its peak effect observed around 48 h post-setting. In summary, White MTA demonstrated the most favorable biocompatibility profile, especially during the initial two days. MTA Fillapex was the most cytotoxic, particularly soon after application, while AH Plus exhibited an intermediate effect.
	Salles L.P. et al. [92]	MTA Fillapex, Epiphany SE, and EndoFill Negative control = Transwell system without material	Stet (24 h)	-	-	24 h, 2 d, 3 d, and 7 d	The MTT assay revealed a significant reduction in cell viability for all tested sealers at 1, 2, and 3 days compared to the control group. By day 7, MTA Fillapex showed a recovery from its initial cytotoxic effect. In contrast, EndoFill and Epiphany SE maintained cytotoxic effects throughout the entire observation period. Notably, Epiphany SE exhibited a time-dependent reduction in cell viability.
	Scelza M.Z. et al. [93]	Real Seal SE, AH Plus, GuttaFlow, Sealapex, Roth 801, and ThermaSeal Plus Negative control = medium	Fresh	24 h, 7 d, 14 d, 21 d, and 28 d	Undiluted	24 h	At the day-1 extraction time point, GuttaFlow exhibited significantly higher cell viability than all other sealers tested. By day 7, GuttaFlow remained significantly different from most groups, with the exception of AH Plus, where no significant difference was found. Unlike at day 1, several other pairwise comparisons between sealers also became statistically significant at this 7-day time point. At day 14, GuttaFlow differed significantly only from Sealapex. However, multiple other significant differences emerged among the other materials, specifically between RealSeal and AH Plus, RealSeal and Thermaseal Plus, RealSeal and Sealapex, AH Plus and Sealapex, Thermaseal and Sealapex, and Sealapex and Roth. No significant differences in cell viability were observed among any of the tested materials at the 21- and 28-day extraction time points.
	Shon W.J. et al. [94]	Apatite Root Sealer Type I, Apatite Root Sealer Type III, CAPSEAL I, CAPSEAL II, and Pulp Canal Sealer EWT Negative control = Transwell system without material	Set (3 h)	-	-	18 h, 24 h, 3 d, 7 d, and 14 d	CAPSEAL I and CAPSEAL II exhibited very low cytotoxicity. In contrast, Apatite Root Sealer Type I and Type III displayed mild cytotoxic effects, which became apparent after 14 days of exposure. Pulp Canal Sealer EWT demonstrated significantly higher cytotoxicity, with observable effects as early as 18 h post-exposure.
	Van Landuyt K.L. et al. [95]	EndoRez, RealSeal, AH Plus Jet, and Calcicur Negative control = medium	Fresh	24 h	1:1, 1:3, 1:10, 1:30, 1:100, and 1:300	24 h	All sealers showed 100% cytotoxicity when undiluted. Cytotoxicity was ranked as follows: AH Plus Jet > Real Seal > EndoREZ > Calcicur.
2011	Loushine B.A. et al. [96]	EndoSequence BC, AH Plus, and Pulp Canal Sealer EWT Negative control = Teflon	Set (AH Plus and Pulp Canal Sealer: 3 d; EndoSequence BC: 10 d)	-	-	24 h, 1 w, 2 w, 3 w, 4 w, 5 w, and 6 w	All tested sealers exhibited severe cytotoxicity at the 24 h time point. Their cytotoxicity profiles, however, diverged significantly over the following six weeks. AH Plus showed a gradual decrease in cytotoxicity, becoming non-cytotoxic as early as the third week. EndoSequence BC Sealer remained moderately cytotoxic up to the fifth week and was only mildly cytotoxic by the sixth week. In contrast, Pulp Canal Sealer EWT maintained severe cytotoxicity throughout the entire six-week observation period.
	Zoufan K. et al. [97]	AH Plus, Tubli-Seal Xpress, GuttaFlow, and Endosequence BC Negative control = medium	Fresh Set (3 d)	24 h and 3 d	300, 600, and 1000 µL	24 h	Fresh sealer eluates: For 1-day eluates, AH Plus was the most cytotoxic. Tubli-Seal was more cytotoxic than Endosequence BC and GuttaFlow at 300 µL and 600 µL. At 1000 µL, AH Plus remained the most cytotoxic, with no significant difference among the other three sealers. For 3-day eluates, AH Plus again showed the highest cytotoxicity. Tubli-Seal was consistently more cytotoxic than Endosequence BC and GuttaFlow across all volumes, between which no differences were observed. Set sealer eluates: For 1-day eluates, both AH Plus and Tubli-Seal were more cytotoxic than Endosequence BC and GuttaFlow. At 300 µL, Tubli-Seal was more cytotoxic than AH Plus. At 600 µL, Tubli-Seal was more cytotoxic than all other sealers. At 1000 µL, Tubli-Seal was more cytotoxic than AH Plus and Endosequence BC but not different from GuttaFlow. For 3-day eluates, AH Plus and Tubli-Seal were more cytotoxic than Endosequence BC and GuttaFlow at 300 µL and 600 µL. At 600 µL, AH Plus exceeded Tubli-Seal in cytotoxicity. At 1000 µL, however, Tubli-Seal was more cytotoxic than AH Plus. Endosequence BC and GuttaFlow showed no significant differences in any set condition.
2010	Al-Hiyasat A.S. et al. [98]	EndoRez, AH Plus, epiphany, and MetaSeal Negative control = medium	Fresh	1 w	Undiluted and 1:10 *v*/*v*	2 d	All tested endodontic sealers reduced the viability of Balb/c 3T3 fibroblasts. In order of increasing cytotoxicity, the ranking was AH Plus (least cytotoxic) < EndoRez < Epiphany < Metaseal (most cytotoxic). For the diluted eluates (1:10 *v*/*v*), AH Plus and Epiphany no longer differed significantly from each other. However, Metaseal remained significantly more cytotoxic than all the other sealers, and EndoRez was significantly more cytotoxic than both AH Plus and Epiphany. Dilution increased cell viability for AH Plus, EndoRez, and Epiphany. In contrast, Metaseal’s cytotoxicity remained similarly high, showing no significant improvement with dilution.
	Bae W.J. et al. [99]	AH 26, Sankin apatite root canal sealer, Pulp Canal Sealer EWT, CAPSEAL I, and CAPSEAL II Negative control = medium	Set (2 d)	3 d	1/2, 1/4, and 1/8	24 h and 14 d	AH26, Pulp Canal Sealer EWT, Sankin, CAPSEAL I, and CAPSEAL II induced cytotoxic effects in HPDLCs after 1 and 14 days. AH26 and Pulp Canal Sealer EWT were more cytotoxic than the other sealers, including Sankin, CAPSEAL I, and CAPSEAL II.
	Ghanaati S. et al. [100]	AH Plus and Gutta Flow Negative control = medium	Fresh	-	-	Alamar Blue: 0 h, 2 h, 6 h, 24 h, 2 d, 3 d, and 4 d Toxilight Kit assay: 24 h	The Alamar Blue assay demonstrated that AH Plus significantly inhibited cell proliferation from 24 h onward compared to the control. In contrast, GuttaFlow exhibited proliferation similar to the control at 24 h, followed by significantly higher proliferation rates at 48, 72, and 96 h. The Toxilight Bioassay revealed significantly higher adenylate kinase release in cells exposed to AH Plus compared to both GuttaFlow and the control, indicating greater cytotoxicity. Conversely, GuttaFlow demonstrated low cytotoxicity and, consistent with the proliferation data, even promoted cell growth over time.
	Huang F.M. et al. [101]	AH 26, Canals, and N2 Negative control = medium	Set (24 h)	24 h	1/2, 1/4, and 1/8	24 h	All three sealers showed concentration-dependent cytotoxicity. AH26 and N2 exhibited equal levels of cytotoxicity and were significantly more cytotoxic than Canals.
	Yu M.K. et al. [102]	AH 26 Negative control = medium	Fresh	24 h, 3 d, 5 d, and 7 d	30%	24 h and 2 d	Cytotoxicity decreased progressively with increasing extraction time. However, a slight increase in cytotoxicity was observed after 48 h of exposure.
	Zhang W. et al. [103]	IRoot SP and AH Plus Negative control = medium	Set (24 h)	24 h	1:1, 1:2, and 1:4	24 h	Undiluted extracts of iRoot SP were non-cytotoxic, while undiluted AH Plus extracts showed slight cytotoxicity. At 1:2 and 1:4 dilutions, however, extracts of both materials were non-cytotoxic.
2009	Ames J.M. et al. [104]	EndoREZ, RealSeal, MetaSEAL, RealSeal SE, and Pulp Canal Sealer Negative control = Teflon	Set (3 d)	-	-	3 d/week (for 5 weeks)	All sealers exhibited severe cytotoxicity immediately after mixing (time 0). Pulp Canal Sealer, EndoREZ, and RealSeal maintained severe cytotoxicity throughout the entire 5-week observation period, showing no significant reduction over time. In contrast, both MetaSEAL and RealSeal SE demonstrated progressive reductions in cytotoxicity, though at different rates. MetaSEAL was severely cytotoxic at week 1, became mildly cytotoxic from weeks 2 to 4, and was non-cytotoxic by week 5. Similarly, RealSeal SE was moderately cytotoxic during weeks 1–2, transitioned to mild cytotoxicity at weeks 3–4, and reached a non-cytotoxic state at week 5.
	Correa G.T. et al. [105]	AH Plus, Fill Canal, and L&C Negative control = medium	Fresh	24 h	100% 10%, 1%, 0.1%, 0.01%, 0.001%, and 0.0001%	24 h	Extracts of AH Plus, Fill Canal, and L&C at 100% and 10% concentrations significantly reduced cell viability compared to the control group. At lower dilutions (1% and below), cytotoxicity was minimal. The only exception was the 1% extract of L&C, which still exhibited significant cytotoxicity.
	Donadio M et al. [106]	Activ GP, RealSeal, AH 26, and Kerr Sealer Negative control = medium	Fresh Set (3 d)	24 h and 3 d	Eluates: 200, 400, 800, and 1200 µL	24 h	Cytotoxicity varied significantly based on the sealer’s setting state, elution time, and extract concentration. For set sealers, AH 26 exhibited the lowest cytotoxicity, particularly at the 200 µL volume in both 1-day and 3-day eluates. In contrast, RealSeal showed the highest cytotoxicity across most tested dilutions (≥400 µL). Among freshly mixed sealers, Kerr Sealer demonstrated the least cytotoxicity at volumes between 400 and 1200 µL. Conversely, AH 26 exhibited the highest cytotoxicity in this state, especially at higher volumes (800–1200 µL). Overall, the cytotoxic effects of the tested materials were dependent on both elution time and extract concentration.
	Gambarini G. et al. [107]	Epiphany SE, Epiphany, and Pulp Canal Sealer Negative control = medium	Set (24 h)	24 h	Undiluted	24 h	All three tested endodontic sealers (Epiphany SE, Epiphany, and Pulp Canal Sealer) exhibited significant cytotoxicity compared to the control group. However, no statistically significant differences in cytotoxicity were observed among the sealers themselves.
	Huang F.M. et al. [108]	AH 26, Canals, and N2 Negative control = medium	Set (24 h)	24 h	1:2, 1:4, and 1:8	2 d	All three materials (AH 26, Canals N2, and N2) were cytotoxic, exhibiting a concentration-dependent effect. Higher extract concentrations consistently led to a greater reduction in cell viability.
2008	Huang F.M. et al. [109]	AH26, Canals, and N2 Negative control = medium	Fresh	24 h	1:2, 1:4, and 1:8	2 d	All tested materials (AH 26, Canals N2, and N2) exhibited concentration-dependent cytotoxicity, where higher extract concentrations resulted in greater cell damage.
	Lodiene G. et al. [110]	AH Plus, EndoREZ, RoekoSeal, Automix, and Epiphany Negative control = medium (extract) and PTFE (filter)	Fresh Set (24 h)	Indirect (set) 24 h	Undiluted	2 h	AH Plus was cytotoxic in its fresh state but became non-cytotoxic after setting for 24 h. In contrast, EndoREZ exhibited no cytotoxicity either immediately after irradiation or in its 24 h set state. RoekoSeal was also non-cytotoxic under both fresh and set conditions. Epiphany, however, was severely cytotoxic when fresh and only moderately cytotoxic after light curing and upon setting. Additionally, extracts of AH Plus and RoekoSeal were rated slightly cytotoxic, EndoREZ remained non-cytotoxic, and Epiphany was confirmed to be severely cytotoxic.
	Pinna L. et al. [111]	MetaSEAL, AH Plus Jet, and Pulp Canal Sealer Negative control = Teflon Positive control = PMMA	Set (3 d)	-	-	3 d/week (for 5 weeks)	All sealers were severely cytotoxic at 72 h (week 0). MetaSEAL remained severely cytotoxic at week 1, mildly cytotoxic at weeks 2–3, and became non-cytotoxic after week 3. AH Plus was moderately cytotoxic at week 1, mildly cytotoxic at weeks 2–3, and also became non-cytotoxic after week 3. In contrast, Pulp Canal Sealer remained severely cytotoxic throughout the entire 5-week period with no significant reduction in toxicity, and at week 5 it was still significantly more cytotoxic than MetaSEAL and AH Plus.
	Valois C.R. et al. [112]	AH Plus, Endofill, and Sealer 26 Negative control = medium	Fresh	24 h	20%, 10%, and 5%	24 h	All root canal sealers (AH Plus, Endofill, and Sealer 26) showed dose-dependent cytotoxicity. At 5% dilution, cell viability remained high. At 10% dilution, a significant reduction in viable cells was observed, and the cytotoxic effect was more pronounced at 20% dilution.
2007	Eldeniz A.U. et al. [113]	Epiphany, EndoREZ, RC Sealer, Acroseal, GuttaFlow, AH Plus, Apexit, and RoekoSeal Negative control = medium	Fresh Set (1 w)	24 h	Undiluted	24 h	Epiphany, EndoREZ, Apexit, and Acroseal were significantly more cytotoxic than the other sealers in both fresh and set conditions. L929 cells were generally more sensitive than HGF cells.
	Merdad K. et al. [114]	Epiphany and AH Plus Negative control = filters with cells and no sealer	Fresh Set (24 h and 2 d)	-	-	2 h	Both AH Plus and Epiphany sealers (freshly mixed) induced moderate cytotoxicity, with AH Plus being significantly more cytotoxic than Epiphany. After 24 and 48 h of setting, all tested materials were non-cytotoxic.
2005	Miletic I. et al. [115]	Roekoseal Automix and AH Plus Negative control = untreated cells	Set (1 h, 24 h, 2 d, 1 w, and 1 m)	-	-	5 d	AH Plus exhibited significantly higher cytotoxicity at setting times of 1 h, 24 h, and 2 days. Its toxicity decreased thereafter, showing no significant difference from the control group at 7 days and 1 month. In contrast, RoekoSeal Automix showed no cytotoxicity at any time point during application. Both the HeLa and L929 cell lines responded similarly under all tested conditions.
2004	Bouillaguet S. et al. [116]	Pulp Canal Sealer, Roeko Seal Automix, Top Seal, and Endo REZ Negative control = Teflon	Fresh Set (24 h)	-	-	24 h, 2 d, and 1 w	Under fresh conditions, only RoekoSeal Automix exhibited low cytotoxicity, with levels comparable to the negative control. The other sealers—Pulp Canal Sealer, TopSeal, and EndoREZ—significantly reduced succinate dehydrogenase (SDH) activity, and their cytotoxicity increased over time, suppressing over 90% of SDH activity after one week. Under set conditions, the overall outcome was similar but more consistent over the observation period. The key exception was EndoREZ, which showed complete suppression of SDH activity as early as 48 h. RoekoSeal Automix again demonstrated only slight, non-significant increases in cytotoxicity.
	Huang T.H. et al. [117]	Sealapex, Canals, and AH Plus Negative control = medium	Fresh Set (24 h)	24 h	0.02, 0.1, 0.5, 2.5, and 12.5 mg/100 mL	24 h	The toxicity of root canal sealers showed a significant dose-dependent effect. The freshly mixed Sealapex exhibited the highest toxicity, which decreased markedly after setting. Canals also showed high initial toxicity that lessened in the set condition. In contrast, AH Plus had moderate toxicity in both fresh and set conditions. These results indicate that toxicity generally decreases as the sealers set, although the extent varies among materials.
2002	Huang T.H. et al. [118]	AH Plus and AH 26 Negative control = medium	Fresh	24 h	0.10, 0.08, 0.04, 0.02, and 0.01 mg/mL	24 h	Both AH26 and AH Plus showed dose-dependent cytotoxicity.
	Schwarze T. et al. [119]	AH Plus, Apexit, Endomethasone, Ketac Endo, and N2 Negative control = medium	Fresh Set (1 h, 5h, and 24 h)	24h	Undiluted	24 h	All sealants tested, except for Apexit, showed cytotoxic effects on both 3T3 fibroblasts and periodontal ligament fibroblasts, with severity influenced by setting time. N2 completely inhibited cell metabolism throughout the entire experimental period, showing the highest toxicity. Endomethasone and Ketac Endo also significantly reduced cell viability at all evaluation times. AH Plus showed only moderate cytotoxicity, limited to the first evaluation times (immediately or 1 h after mixing), while the eluates obtained at 5 and 24 h were not toxic. Apexit showed no cytotoxicity.
2001	Huang T.H. et al. [120]	Canals, Sealapex, AH 26, and AH Plus Negative control = medium	Fresh	24 h	0.01, 0.025, 0.04, 0.05, and 0.10%	1 h	All tested root canal sealers caused significant LDH loss in a concentration-dependent manner, indicating membrane damage and cytotoxicity. The highest LDH release was observed with AH 26 at a concentration of 0.10%, followed by Canals at a concentration of 0.05%, Sealapex at a concentration of 0.10%, and AH Plus at a concentration of 0.10%. Among the materials, AH Plus showed the lowest cytotoxicity, while AH 26 showed the highest.
2000	Azar N.G. et al. [121]	AH 26, AH Plus, and Zinc Oxide Eugenol Negative control = medium	Fresh	1 h, 4 h, 8 h, 24 h, 2 d, 5 d, 1 w, 2 w, 4 w, and 5 w	Undiluted	22 h	Zinc Oxide Eugenol exhibited strong and persistent cytotoxicity, though its early effects (0–1 h) were less pronounced. AH26 caused a severe early cytotoxic effect lasting up to 1 week, with a notable reduction thereafter, though toxicity remained significant. AH Plus showed early cytotoxicity (1–4 h), but no effects were detectable beyond 4 h.
	Huang T.H. et al. [122]	AH 26 and AH Plus Negative control = medium	Fresh	24 h	0.01, 0.04, and 0.1%	4 h, 10 h, and 24 h	AH26 caused significant LDH leakage in hepatocytes at concentrations above 0.04%, while AH Plus was only significantly cytotoxic at concentrations above 0.1%. Both sealers induced increasing LDH leakage over 4, 10, and 24 h when applied at 0.04% and 0.1% concentrations. AH26 consistently caused higher LDH release than AH Plus, especially at 0.1%, indicating greater cytotoxicity. Time-dependent increase in LDH leakage was induced when cells were treated with 0.04 and 0.1% AH26 and AH Plus.
1999	Telli C. et al. [123]	Sankin apatite root canal sealers I, Sankin apatite root canal sealers II, Sankin apatite root canal sealers III, Calciobiotic Root Canal Sealer, Ketac Endo, AH26, and Endomethasone Negative control = medium	Set (1 h)	1 h	1/2, 1/4, 1/8, and 1/16	24 h	Cytotoxicity tests showed that SARCS 1-3 and Calciobiotic Root Canal Sealer did not exhibit cytotoxicity at any dilution. In contrast, AH26, Ketac Endo, and Endomethasone exhibited varying degrees of cytotoxicity.
1997	Beltes P. et al. [124]	Ketac Endo and Endion Negative control = medium	6 h	-	-	24 h, 2 d, and 3 d	Endion was significantly more cytotoxic than Ketac-Endo at all time points.

**Abbreviations**: EWT, Extended Working Time; BC, bioceramic; (h), hours; (d), days; (w), weeks. Cell lines: hDPSC, human dental pulp stem cell; hDPC, human dental pulp cell; SAOS-2, human osteoblastic cells; HeLa, HeLa human cervical cancer cells; hGF, human gingival fibroblast; hMSC, immortalized human bone marrow-derived mesenchymal stem cell; hPDLF, human periodontal ligament fibroblast; hOB, human osteoblast; DF-MSC, dental follicle mesenchymal stem cell; hPDLC, human periodontal ligament cell; hPDLSC, human periodontal ligament stem cell; hSCAPs, human apical papilla stem cells; hTGSCs, human tooth germ stem cells; OC2, human oral cancer cell line; THP-1, human monocytic cells; U2OS, human osteoblastic cell line; MG63, human osteoblast-like cells; BMMs, bone marrow-derived monocyte–macrophages; rSCAPs, rat apical papilla stem cells; RAW 264.7, RAW 264.7 mouse macrophages; L929, L929 mouse fibroblasts; MC3T3-E1, mouse osteoblast-like cells; NIH/3T3, NIH/3T3 mouse fibroblasts; V79, Chinese hamster fibroblasts; ROS 17/12.8, ROS 17/12.8 rat osteosarcoma cells; BHK21/C13, Baby Hamster Kidney.

#### 3.3.1. Epoxy Resin-Based Sealers

The most extensively investigated sealer was the epoxy resin-based material AH Plus, evaluated in 53 studies. In the vast majority of these investigations, AH Plus exhibited cytotoxic effects under all tested conditions [28,29,32,37,41,43,48,51,52,53,54,60,62,63,64,65,66,72,74,75,76,79,80,82,88,90,91,93,97,98,100,105,112,113,117,118,119,120,121,122]. Only two studies reported AH Plus to be non-cytotoxic [50,57], while two others documented the absence of cytotoxicity exclusively at low concentrations [40,103]. Evidence regarding the temporal pattern of cytotoxicity was heterogeneous: one study showed complete cell death after just 24 h of exposure [69], whereas another observed the same effect after 3 days [67]. By contrast, one study reported that after 7 days of exposure, AH Plus did not differ significantly from the negative control [44], and another found a comparable outcome only after 3 weeks [96]. Two studies indicated that AH Plus was cytotoxic in its freshly mixed state but became non-cytotoxic after 7 days of setting [81,115]; conversely, two other studies found it to be non-cytotoxic after just 24 h of setting [110,114]. Additionally, one investigation reported that AH Plus remained consistently cytotoxic when tested undiluted but exhibited cytotoxicity only in its fresh condition when diluted [85].

The second most extensively studied epoxy resin-based sealer was AH 26, evaluated in 11 studies, all of which reported it as cytotoxic [99,101,102,106,108,109,118,120,121,122,123]. AH Plus Jet was investigated in 12 studies and was similarly found to be cytotoxic in most of them [32,35,41,68,71,87,89,95]. However, Santiago M.C. et al. and Chen J. H. et al. reported AH Plus Jet as biocompatible [31,36], and Martinho F. C. et al. indicated that it was non-cytotoxic when tested at low concentrations [70]. Pinna L. et al. found it to be non-cytotoxic after 3 weeks of exposure [111].

Other epoxy resin-based sealers, such as Acroseal, SimpliSeal, TopSeal, Sealer Plus, ThermaSeal Plus, Dia-Proseal, and AD Seal, also demonstrated varying degrees of cytotoxicity [53,64,73,75,82,93,113,116].

#### 3.3.2. Methacrylate Resin-Based Sealers

All methacrylate resin-based sealers, EndoREZ, Epiphany, Epiphany SE, RealSeal, RealSeal SE, RealSeal XT, MetaSEAL, MetaSEAL Soft, and SuperBond Sealer, demonstrated some degree of cytotoxicity across the included studies [49,55,70,71,74,85,87,88,92,93,95,98,104,106,107,110,111,113,114]. However, several investigations also reported conditions under which these materials exhibited limited or no cytotoxic effects. Specifically, EndoREZ and RealSeal were found to be non-cytotoxic at low concentrations in one study [70]. Ames J.M. et al. documented that both RealSeal SE and MetaSEAL ceased to exhibit cytotoxicity after 5 weeks of continuous cell exposure [104]. Lodiene G. et al. indicated that EndoREZ was non-cytotoxic not only in its freshly mixed state but also after a 24 h setting period [110]. Similarly, one study showed that Epiphany was non-cytotoxic when freshly mixed and remained non-cytotoxic after 24 h of setting [114]. RealSeal XT was reported to be non-cytotoxic in one investigation [87], while Pinna L. et al. found that MetaSEAL no longer displayed cytotoxic effects after 3 weeks of exposure [111].

#### 3.3.3. Glass Ionomer Cements

The glass ionomer cements Ketac Endo, Activ GP, Nishika Canal Sealer BG, and Endion were consistently reported as cytotoxic in all studies in which they were evaluated [49,106,119,123,124]. These findings indicate a uniform cytotoxic profile across this material class, with no study documenting conditions under which these sealers exhibited reduced or absent cytotoxicity.

#### 3.3.4. Silicone Based Sealers

The silicone-based sealers evaluated in the included studies were GuttaFlow [71,93,97,100,113], GuttaFlow 2 [49,51,53,66,72,76,88], GuttaFlow Bioseal [50,66,76], RoekoSeal [85,113], and RoekoSeal Automix [55,110,115,116]. Overall, these materials demonstrated a favorable biocompatibility profile in most investigations. Zoufan K. et al. reported that GuttaFlow Bioseal exhibited significantly lower cytotoxicity compared with an epoxy resin-based sealer (AH Plus) and a zinc oxide–eugenol-based sealer (Tubli-Seal), while showing no statistically significant differences from a bioactive sealer (EndoSequence BC) under the tested conditions [97]. Ghanaati S. et al. observed that GuttaFlow did not differ significantly from the control during the first 24 h; however, after 48 and 72 h, it promoted a higher proliferation rate than the control and displayed significantly lower cytotoxicity compared with AH Plus [100]. In contrast, one investigation found that GuttaFlow 2 caused a significant reduction in cell viability after a 72 h elution period at both low and high concentrations, and after a 24 h elution period when tested at high concentrations [88]. Another study reported that GuttaFlow Bioseal exhibited cytotoxicity only during the first 24 h and exclusively at high concentrations, both in hPDLFs and hOBs [50]. Finally, Pawinska M. et al. documented that RoekoSeal Automix demonstrated cytotoxic effects [55].

#### 3.3.5. Calcium Hydroxide-Based Sealers

Sealapex, Apexit, Apexit Plus, Sealer 26, L&C, and Calciobiotic Root Canal Sealer were reported to be cytotoxic in most of the studies in which they were evaluated [37,55,71,74,93,105,112,113,117,120,123]. Nonetheless, isolated findings suggest variability within this material class. Chang S.W. et al. documented a good biocompatibility profile for Sealapex [86], while two studies reported similarly favorable results for Apexit [70,119]. With respect to Sealer 26, the evidence was conflicting: Oliveira P. Y. et al. found it to be non-cytotoxic [45], whereas another study reported cytotoxic effects when the material was tested at high concentrations [112].

#### 3.3.6. Zinc Oxide–Eugenol-Based Sealers

Among zinc oxide–eugenol-based sealers, Pulp Canal Sealer was the most extensively investigated, being evaluated in eight studies, and was found to be cytotoxic in most of them under all tested conditions [61,62,104,107,111,116]. Jung S. et al. reported cytotoxicity after 2 days of cell exposure, whereas another found the material to be non-cytotoxic when tested at low concentrations [69]. Pulp Canal Sealer Extended Working Time was assessed in four studies and was consistently cytotoxic in all of them [80,94,96,99]. N2, also reported in four studies, demonstrated cytotoxicity in every investigation [101,108,109,119]. Endofil was evaluated in four studies: two reported cytotoxicity under all tested conditions [75,92], one documented cytotoxicity only after 2 days of exposure [45], and another found the material to be non-cytotoxic at low concentrations [112]. Canals, likewise, analyzed in four studies, was consistently cytotoxic [101,108,109,120]. Endométhasone was also reported in four studies and demonstrated cytotoxicity in each of them [55,80,119,123]. Finally, Roth’s Sealer, Zinc Oxide–Eugenol, Tubli-Seal, Tubli-Seal Xpress, Pulpdent Root Canal Sealer, and FillCanal were cytotoxic in all studies in which they were evaluated [53,71,72,74,84,93,97,105,121].

#### 3.3.7. Bioactive Sealers

Bioactive sealers were evaluated in 67 studies, making them the second most extensively investigated material category. Among them, MTA Fillapex was the most frequently studied and was consistently reported as cytotoxic across all available investigations [33,37,46,56,62,63,66,69,71,72,73,76,79,80,81,82,84,86,89,91,92]. Endoseal MTA also generally demonstrated a reduction in cell viability [59,64,77], although one study documented a favorable biocompatibility profile [67]. MTA Angelus and AGM MTA were reported to be cytotoxic at high concentrations in a single study [30], whereas another investigation found MTA Angelus to be cytocompatible in both fresh and set conditions [91], i-MTA SP demonstrated a dose-dependent response and a time-dependent increase in cytotoxicity [27]. NEOMTA Plus showed good biocompatibility in both pulp cells and osteoblasts [33]. BrightEndo MTA was cytotoxic during the first 24 h, but its cytotoxicity progressively decreased thereafter, ultimately promoting increased cell viability after 7 days [54]. MTA Plus was reported as non-cytotoxic [84]. ProRoot MTA generally showed a favorable cytocompatibility profile and even increased cell viability [78], although it was found to be cytotoxic at high concentrations in one study [88]. Apatite Root Sealer consistently reduced cell viability [86,94,99], except in one investigation where it demonstrated good biocompatibility [123].

BioRoot RCS exhibited good biocompatibility in most studies [36,42,61,62,67,68,69,72,73,77,83], although one study reported cytotoxicity [50]. Increased cell viability was observed at low concentrations in two studies [6,42] and even at higher concentrations in another [77]. Conversely, three studies reported cytotoxicity when the material was tested undiluted [62,68,69]. Endosequence BC was analyzed in 12 studies and was generally non-cytotoxic [35,47,48,59,60,67,68,72]. Increased cell viability was reported after 3 days at a 1:2 concentration in one study [59] and after 24 h at high concentrations in another [60], while Ye Y et al. showed a significant increase in cell viability after 3 days when analyzed at the 1:64 dilution compared with the negative control [35]. Two studies found it to be non-cytotoxic except at high concentrations [58,63], while one study reported cytotoxicity [96]. Endosequence BC HiFlow demonstrated increased cell viability in two studies [44,60], was cytotoxic in one, [31] and non-cytotoxic except at high concentrations in another [58].

TotalFill BC generally exhibited good biocompatibility [37,41,57,79]. In two studies, it was cytotoxic when undiluted but non-cytotoxic when diluted [38,65], and in one of these investigations dilution resulted in increased cell viability [65]. Wuersching S. N. et al. found TotalFill BC to be non-cytotoxic on hPDLFs but cytotoxic at high concentrations on hOBs [50]. IRoot SP displayed good cytocompatibility in most studies, both in undiluted and diluted forms [27,34,44,52,86,103]. Increased cell viability was observed at a 1:4 dilution after 7 days in one study [44] and Kwan DCY et al. reported significant cytotoxicity at the 1:1 dilution but also an increase in the viability of cells exposed for 3 days to the 1:10 dilution of the eluate [29], while Wang Z et al. showed an increase in cell viability after 24 h of exposure to the eluate [34]. However, Zhou G. et al. reported cytotoxicity at high concentrations [40], while Guven E.P. et al. observed cytotoxicity only after 5 days of exposure [89]. IRoot BP Plus and IRoot FS increased cell viability when tested in set conditions at both low and high concentrations after 24 h, 2 days, and 3 days [78]. Bio-C Sealer was reported as cytocompatible in three studies [33,56,65], with Pedrosa M. D. S. et al. showing a significant increase in cell viability after 3 days at a 1:16 concentration [56]. Ramos RF et al. showed a reduction in cell viability when Bio-C Sealer and Bio-C Sealer Ion^+^ were analyzed at the 1:1 dilution and reported good cytocompatibility at higher dilutions [32]. Two studies found it to be cytotoxic when undiluted but non-cytotoxic when diluted [42,46]. In contrast, one investigation reported cytotoxicity [31], and another found cytotoxicity after 2 days of exposure [47].

AH Plus Bioceramic was non-cytotoxic in four studies [35,36,38,41], while three studies found it to be cytotoxic when undiluted but non-cytotoxic when diluted [29,39,48]. Santiago M.C. et al. reported cell type-dependent effects, with cytotoxicity on hDPCs but not on SAOS-2 cells [33]. Sealer Plus Bioceramic reduced cell viability at 1:1 and 1:2 concentrations in two studies [42,57], was cytotoxic after 2 days in one investigation [47], and increased cell viability at a 1:5 concentration in another [42]. CeraSeal increased cell viability in three studies [43,54,59] and was non-cytotoxic in one [28]. Neosealer Flo was non-cytotoxic in one study [28], but cytotoxic at high concentrations in another [38]; Chen Z et al. reported good cytocompatibility and observed both a dose-dependent response and a time-dependent increase in cytotoxicity [27], while Wang Z et al. showed significantly enhanced cell proliferation compared with the control group when cells were analyzed at high dilutions after two days of exposure [34]. BioRoot Flow demonstrated good cytocompatibility in two studies [36,39]. CRoot SP showed good cytocompatibility [27,29,40] and Kwan DCY et al. reported an increase in the viability of cells exposed for 3 days to the 1:10 dilution of the CRoot eluate [29], while Zhou G. et al. reported cytotoxicity only at high concentrations and after 3–5 days of exposure [40]. Cimmo HP was cytotoxic when undiluted but demonstrated good cytocompatibility at low concentrations [46,56]; moreover, at a 1:16 dilution, it increased cell viability after 48 h in one study [56]. Endoseal TCS showed good cytocompatibility and increased cell viability after 7 days when tested fresh [54]. Wellroot ST demonstrated good biocompatibility and increased cell viability after 3 days [64]. Finally, Nano-Ceramic Sealer exhibited good cytocompatibility in one study [64] but was found to be cytotoxic in another [77].

### 3.4. Effect of Setting Time on Cytotoxicity

In studies that evaluated materials at different setting times, most reported that set sealers were less cytotoxic than freshly mixed ones [30,35,53,54,64,71,72,81,85,88,91,110,114,116,117,119]. Erdogan H. et al. specifically noted that setting time influenced the material’s cytotoxicity [52], while Pawinska M. et al. indicated that variations in setting affected cytotoxicity, although not in a statistically significant manner [55].

### 3.5. Effect of Extraction Time on Cytotoxicity

Among the studies that evaluated materials using tests on extracts obtained from eluates prepared at different extraction times, Chen J. H. et al. and Park M. G. et al. reported no influence of extraction time on material cytotoxicity [36,54]. Wuersching S. N. et al. found that materials exhibited a better biocompatibility profile after 7 days of extraction compared with 24 h [50], whereas Vouzara T. et al. reported higher cytotoxicity after 7 days relative to 24 h [73]. In addition, Lee J. K. et al. demonstrated that materials improved biocompatibility when evaluated after 3 days of extraction rather than after 2 days [64]. Three studies indicated that extraction time modulated cytotoxicity, either increasing or decreasing it depending on the conditions tested [88,97,106]. Another three studies observed a progressive improvement in biocompatibility as the eluate extraction time increased [93,102,121].

### 3.6. Effect of Extract Concentration on Cytotoxicity

In studies assessing the influence of different eluate concentrations, most findings indicated that higher concentrations were associated with increased cytotoxicity for several materials [27,28,29,30,31,32,34,35,37,40,41,42,46,48,50,52,57,58,59,63,68,70,75,84,85,88,91,95,98,101,103,105,106,108,109,112,117,118,120,122,123]. Manda P. et al. specifically showed a concentration-dependent reduction in biocompatibility for ProRoot MTA and GuttaFlow 2, whereas AH Plus and RealSeal remained consistently cytotoxic irrespective of concentration [88]. Two investigations found no correlation between eluate concentration and cytotoxicity [60,79]. Other studies reported that BioRoot, Sealer Plus Bioceramic, and TotalFill BC decreased cell viability at high concentrations but significantly increased viability at lower concentrations compared with the control [42,57,65]. Lee B. N. et al. found that AH Plus maintained high cytotoxicity regardless of dilution [63], whereas Lv F. et al. reported that undiluted eluates of iRoot FS, iRoot BP, and ProRoot MTA resulted in higher biocompatibility than their diluted forms [78]. In other studies, iRoot SP, CRoot, NeoSealer Flo, and EndoSequence BC Sealer induced a significant increase in cell viability compared with the negative control when analyzed at high dilutions [29,34,35].

### 3.7. Effect of Cell Exposure Time on Cytotoxicity

Among the studies that evaluated the cytotoxicity of materials at different cell exposure times, considerable heterogeneity emerged regarding the effect of exposure duration on cytotoxic behavior. Several studies reported that longer exposure to certain materials was associated with increased cytotoxicity [28,30,31,40,41,45,47,53,58,59,61,62,64,69,73,76,77,79,87,89,92,94,102,104,116,122]. Cotti et al. observed a progressive reduction in cell viability over time for AH Plus Jet, whereas the cytocompatibility of RealSeal XT appeared unaffected by exposure duration [87]. Salles et al. reported that Epiphany SE caused a time-dependent decrease in cell viability, while MTA Fillapex reduced viability during the first 3 days of exposure, followed by recovery at 7 days [92].

Ames et al. found a time-dependent reduction in cytotoxicity for MetaSEAL and RealSeal SE and reported that exposure time did not influence the cytotoxicity of Pulp Canal Sealer, EndoREZ, or RealSeal [104]. Pinna et al. similarly demonstrated a reduction in cytotoxicity over time for MetaSEAL and AH Plus Jet, but noted that the cytotoxicity of Pulp Canal Sealer was unaffected by exposure duration [111].

Seven studies showed that, for some sealers, longer exposure was associated with increased cell viability [54,61,62,65,69,96,100]. Park et al. observed that CeraSeal and EndoSeal TCS, which showed no significant differences compared with the control after 24 h, 2 days, and 3 days, produced a statistically significant increase in viability at 7 days [54]. Jeanneau et al. reported increased viability in cells exposed to BioRoot RCS after 6 and 9 days, while noting a significant reduction in viability for cells treated with Pulp Canal Sealer over the same periods [61].

Jung et al. found a time-dependent decrease in cell viability for MTA Fillapex, Pulp Canal Sealer, and AH Plus, but an increase for BioRoot RCS [62], with similar findings in another study by the same authors (excluding MTA Fillapex) [69]. Loushine et al. reported that EndoSequence Bioceramic Sealer and AH Plus were strongly cytotoxic during the first 24 h but showed reduced cytotoxicity over time; conversely, Pulp Canal Sealer EWT remained cytotoxic even after 6 weeks of exposure [96].

Four studies reported that exposure duration did not influence the cytotoxicity of the tested materials [39,51,60,86].

### 3.8. Risk of Bias Assessment of the Included Studies

**Figure 2:**
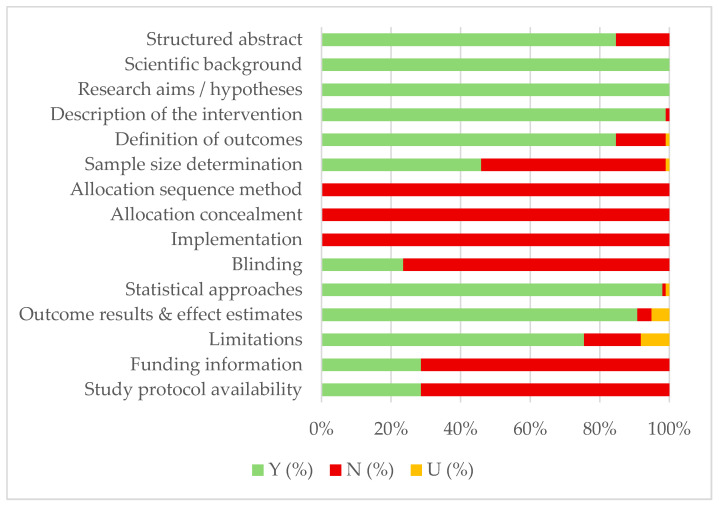
Assessment of the methodological quality of included in vitro studies.

A total of 98 in vitro studies were evaluated for methodological quality using the modified CONSORT-based checklist for preclinical dental materials research proposed by Faggion Jr. [26] The detailed scoring for each checklist item (Items 1–15) is reported in Table S3 and is summarized schematically in Figure 2.

Overall, reporting quality varied substantially across studies, with none of the included investigations fulfilling all 15 checklist items.

Across the dataset, items related to title/abstract structure (Item 1), scientific background (Item 2a), study objectives (Item 2b), intervention description (Item 3), outcome definition (Item 4), and sample size reporting (Item 5) were consistently well-reported, with many studies scoring “Yes” for these domains. By contrast, randomization procedures (Items 6–8) and blinding (Item 9) were almost universally absent, with many studies scoring “No,” reflecting the common lack of allocation procedures in in vitro experimental designs.

Statistical methods (Item 10) and outcome reporting with effect estimates (Item 11) were generally described, although the level of detail varied. Items related to limitations (Item 12) and funding disclosures (Item 13) were moderately reported, whereas protocol availability (Item 14) was seldom included, indicating limited transparency regarding experimental preregistration or methodological protocols.

Based on the total number of checklist items adequately fulfilled, studies were stratified into three methodological quality categories: 91 studies demonstrated a moderate risk of bias, fulfilling between 6 and 10 items; 2 studies satisfied 11 or more items and were therefore classified as having a low risk of bias; and 5 studies reported fewer than six items and were rated as having a high risk of bias. These findings reflect substantial heterogeneity in adherence to recommended methodological standards for in vitro research on dental materials.

## 4. Discussion

The present systematic review provides a comprehensive, updated, and structured synthesis of the available in vitro evidence concerning the cytotoxicity of root canal sealers that have been commercially marketed over the years. By including 98 in vitro studies published over more than two decades, this review highlights both the extensive scientific interest in the biological behavior of endodontic sealers and the substantial methodological heterogeneity that currently limits data comparability and precludes quantitative synthesis.

Across the included studies, all categories of root canal sealers demonstrated some degree of cytotoxicity, particularly when tested in their freshly mixed state, at higher extract concentrations, or after prolonged exposure times. This finding was consistently reported for epoxy resin-based, zinc oxide–eugenol-based, glass ionomer, silicone-based, calcium hydroxide-based, methacrylate resin-based, and bioactive sealers, confirming that no endodontic material can be considered biologically inert under in vitro conditions [28,29,32,37,40,45,61,69,80,119]. These observations are consistent with the broader literature on dental biomaterials, which emphasizes that cytotoxic effects are frequently detected when materials are evaluated under simplified experimental conditions [125,126]. From a clinical perspective, these findings reinforce the fundamental principle that apical extrusion of sealers should be minimized, as direct or indirect contact with periapical tissues may elicit unfavorable biological responses.

Despite this general observation, relevant differences emerged among material classes. In several studies included in this review, bioactive sealers generally demonstrated a more favorable cytotoxicity profile, in several but not all experimental conditions, when compared with conventional resin-based or zinc oxide–eugenol-based sealers [27,28,29,31,32,33,34,35,36,38,39,40,41,42,43,44,47,50,57,60,62,65,66,67,68,69,72,73,77]. Calcium silicate-based materials such as BioRoot RCS, EndoSequence BC, iRoot SP, TotalFill BC, CeraSeal, Bio-C Sealer, and related formulations frequently showed cell viability values comparable to negative controls, particularly when tested after complete setting and at diluted extract concentrations across different cellular models, including periodontal ligament fibroblasts, osteoblasts, and mesenchymal stem cells [36,42,50,61,67,68,69]. These findings agree with previous reports suggesting improved biocompatibility of calcium silicate-based sealers compared with traditional endodontic materials [127,128,129].

However, the biological behavior of bioactive sealers was not uniformly non-cytotoxic. Several studies included in the present review reported measurable cytotoxic effects associated with specific bioceramic formulations, especially under unfavorable experimental conditions such as testing freshly mixed materials, using undiluted extracts, or evaluating early exposure time points [31,32,33,46,56,59,71,76]. These discrepancies likely reflect differences in material composition, setting reactions, ion release kinetics, alkalinity, and experimental protocols rather than true contradictions in biological behavior. Consequently, although bioactive sealers generally exhibit a more favorable cytotoxicity profile, they should not be indiscriminately considered biologically inert.

A particularly relevant finding with potential clinical implications is the time-dependent improvement in cell viability observed for several bioactive sealers. Multiple studies reported increased cell viability over time for calcium silicate-based materials compared with traditional sealers, in some cases approaching or even exceeding control values [28,29,30,34,37,50,57,65]. This phenomenon has been attributed to the gradual release of calcium and hydroxyl ions, which may enhance cellular metabolic activity, promote proliferation, and create an alkaline microenvironment favorable to cell survival and mineralization-related processes [130,131]. In this context, recent in vitro investigations have demonstrated that ion release from bioactive dental materials is strongly influenced by environmental conditions such as pH and temperature, which in turn may modulate cellular responses and biological performance over time [132]. Similar findings have been reported for fluoride- and calcium-releasing restorative materials, further supporting the concept that ionic exchange plays a key role in the biological behavior of bioactive dental materials under laboratory conditions [133,134]. Nevertheless, these findings were observed under specific in vitro conditions and should not be interpreted as direct evidence of clinical bioactivity.

In contrast, zinc oxide–eugenol-based sealers and glass ionomer sealers generally exhibited the highest cytotoxic potential among the materials analyzed [45,49,53,69,74,80,101,108,109,119,121,122,123,124]. The release of eugenol and acidic components has been proposed as a major contributor to reduced cell viability, oxidative stress, and membrane damage [133]. However, it must be acknowledged that these conclusions are based on a relatively limited number of studies compared with other material classes, representing an important limitation when interpreting the relative cytotoxic ranking of these sealers.

Among resin-based materials, AH Plus emerged as the most extensively investigated sealer, being included in the majority of the analyzed studies [28,29,32,37,40,41,43,44,48,50,51,52,53,54,57,60,62,63,64,65,66,67,69,72,74,75,76,79,80,81,82,85,88,90,91,93,96,97,98,100,103,105,110,112,113,114,115,117,118,119,120,121,122]. While AH Plus frequently demonstrated acceptable biocompatibility after complete setting and at lower extract concentrations, most studies reported some degree of cytotoxicity under at least one experimental condition. Notably, only two studies included in this review described AH Plus as non-cytotoxic, underscoring the strong influence of experimental parameters such as setting time, dilution, and exposure duration on biological outcomes [29,90]. These findings are consistent with previous reviews highlighting the condition-dependent cytotoxicity of epoxy resin-based sealers [125,135].

### 4.1. Limitations of the Study

The findings of this systematic review should be interpreted in light of several limitations inherent to both the included studies and the review design itself. The absence of a quantitative meta-analysis represents a major methodological limitation of this study. The included studies exhibited pronounced heterogeneity with respect to cytotoxicity assays (e.g., MTT, XTT, Alamar Blue, CCK-8, Neutral Red, and LDH release), cell types (fibroblasts, osteoblasts, stem cells, immune cells, and cancer-derived lines), test procedures, setting times, extraction procedures, extract concentrations, and exposure durations. In addition to differences in biological testing protocols, variability in the physicochemical behavior of dental materials during setting and polymerization may also contribute to heterogeneous experimental outcomes, as demonstrated by computational studies highlighting material-dependent stress development and structural behavior under simulated conditions [136]. In this context, it is worth noting that the variability in cytotoxicity outcomes may also be partially attributed to differences among cell viability assays themselves. A systematic review by Pintor et al. demonstrated that tetrazolium salt-based assays, including MTT and XTT, generally show moderate to good concordance with other cell viability tests when evaluating root canal filling materials, although discrepancies may occur depending on the cell type and experimental conditions [137]. These findings support the reliability of tetrazolium-based assays for cytotoxic screening, while simultaneously reinforcing the need for multiparametric and standardized testing protocols to improve the comparability and translational relevance of in vitro data.

Furthermore, intrinsic differences in cellular sensitivity and metabolic activity among the tested cell lines likely contributed to the observed variability in cytotoxic responses. Similar methodological limitations have been reported in previous studies addressing the biological assessment of endodontic materials [126,138,139,140,141,142]. Collectively, these factors precluded meaningful quantitative synthesis and direct numerical comparison of outcomes across studies. A previous review of in vitro and in vivo studies explored related aspects of the biological behavior of endodontic sealers under controlled experimental conditions, further supporting the need for a comprehensive and systematic synthesis of the available evidence [143].

An additional methodological aspect that warrants careful consideration concerns the evaluation of freshly mixed sealers. According to ISO 10993-12, ISO 10993-6, and ISO 6876:2012, biological testing of endodontic sealers should ideally be performed on set materials under standardized conditions, as setting significantly influences material stability and biological behavior [144,145,146]. Nevertheless, a substantial number of in vitro studies included in this review investigated freshly mixed sealers, often with the aim of simulating early tissue exposure following accidental apical extrusion. While such data may provide insight into worst-case or early-exposure scenarios, results obtained from fresh materials should be interpreted with caution and cannot be considered fully representative of long-term clinical biocompatibility. This methodological heterogeneity further supports the need for standardized testing protocols and careful contextualization of in vitro findings.

From a clinical standpoint, the findings of this systematic review suggest that bioceramic and bioactive sealers present the most favorable in vitro cytotoxicity profiles among currently available materials, supporting their increasing use in contemporary endodontic practice, particularly in clinical scenarios where material extrusion may occur. It should also be acknowledged that periapical healing is a multifactorial process influenced not only by the biological properties of endodontic materials, but also by host-related and adjunctive biological factors, as highlighted in recent clinical systematic reviews on regenerative and platelet-based approaches in endodontic surgery [147,148]. Nevertheless, given the inherent limitations of in vitro models, these results should be interpreted with caution. Therefore, these results should be interpreted as supportive, rather than definitive, when guiding clinical material selection.

### 4.2. Future Research Directions

Future research should focus on the development and adoption of standardized in vitro testing protocols to improve the comparability and reproducibility of cytotoxicity assessments across different endodontic sealers. Greater consistency in cell models, assay selection, extract preparation, and exposure parameters would facilitate more robust synthesis of evidence.

Moreover, translational studies integrating advanced in vitro models, such as three-dimensional cell cultures or co-culture systems, as well as in vivo and clinical investigations, are needed to better elucidate the biological behavior of sealers under clinically relevant conditions. Long-term studies evaluating chronic exposure, material degradation, and inflammatory responses following sealer extrusion would further enhance clinical applicability. Finally, future investigations should aim to correlate cytotoxicity findings with clinical outcomes, supporting evidence-based material selection and optimizing patient-centered endodontic care.

## 5. Conclusions

This systematic review demonstrates that all commercially available root canal sealers exhibit some degree of cytotoxicity in vitro, with considerable variability depending on material composition, experimental design, and testing conditions. Bioactive and bioceramic sealers generally showed a more favorable cytotoxicity profile compared with traditional materials, particularly after complete setting and at diluted extract concentrations. Several studies also reported time-dependent improvements in cell viability for calcium silicate-based sealers, suggesting a potentially more supportive biological behavior under specific in vitro conditions.

However, these findings were not consistent across all experimental settings, and measurable cytotoxic effects were also reported for certain bioceramic formulations. Zinc oxide–eugenol-based and glass ionomer sealers tended to display higher cytotoxic potential, although the limited number of available studies warrants cautious interpretation. Resin-based sealers, particularly AH Plus, remain extensively studied and clinically successful, yet their biological behavior appears to be strongly influenced by experimental variables.

The marked heterogeneity among the included studies precluded quantitative synthesis and underscores the urgent need for standardized in vitro protocols for cytotoxicity assessment of endodontic sealers. From a clinical perspective, cytotoxicity should be considered a relevant parameter when selecting endodontic sealers, especially in situations where material extrusion or prolonged tissue contact may occur. Further standardized in vitro, in vivo, and clinical studies are required to confirm the translational relevance of these results and to support evidence-based decision-making in endodontic practice.

## Figures and Tables

**Table 1:** Search strategy.

Database	Search Strategy	Hits
**Medline** (via PubMed)	((Root Canal Filling Materials OR root canal sealer OR root canal filling OR root canal obturation OR Epoxy Resins OR Zinc Oxide-Eugenol Cement OR Glass Ionomer Cements OR mineral trioxide aggregate OR bioceramic sealer OR calcium silicate cements OR Endodontics sealer OR hydraulic cement sealers OR silicone cement OR salicylate cement OR bioactive endodontic sealer OR tricalcium silicate cement) AND (cytotoxicity OR cell viability OR Cell death OR Cell survival OR Toxicity tests))	**1655**
**Scopus**	(TITLE-ABS-KEY (root AND canal AND filling AND materials) OR TITLE-ABS-KEY (root AND canal AND sealer) OR TITLE-ABS-KEY (root AND canal AND filling) OR TITLE-ABS-KEY (root AND canal AND obturation) OR TITLE-ABS-KEY (epoxy AND resins) OR TITLE-ABS-KEY (zinc AND oxide-eugenol AND cement) OR TITLE-ABS-KEY (glass AND ionomer AND cements) OR TITLE-ABS-KEY (mineral AND trioxide AND aggregate) OR TITLE-ABS-KEY (bioceramic AND sealer) OR TITLE-ABS-KEY (calcium AND silicate AND cements) OR TITLE-ABS-KEY (endodontics AND sealer) OR TITLE-ABS-KEY (hydraulic AND cement AND sealers) OR TITLE-ABS-KEY (silicone AND cement) OR TITLE-ABS-KEY (salicylate AND cement) OR TITLE-ABS-KEY (bioactive AND endodontic AND sealer) OR TITLE-ABS-KEY (tricalcium AND silicate AND cement) AND TITLE-ABS-KEY (cytotoxicity) OR TITLE-ABS-KEY (cell AND viability) OR TITLE-ABS-KEY (cell AND death) OR TITLE-ABS-KEY (cell AND survival) OR TITLE-ABS-KEY (toxicity AND tests))	**2391**
**Cochrane Oral Health Group Databases**	(Cytotoxicity of root canal filling materials); ti,ab,kw	**10**

**Table 2:** Population, Intervention, Comparison and Outcome (PICO) strategy used for assessment of scientific literature.

Parameter	Assessment
Population (P)	Cell models
Intervention (I)	Sealer specimens or sealer extract
Comparison (C)	Other root canal sealers or non-exposed control groups
Outcome (O)	Cytotoxicity (measured as cell viability, cell death, or proliferation)

## Data Availability

The original contributions presented in this study are included in the article/Supplementary Materials. Further inquiries can be directed to the corresponding authors.

## Supplementary Materials

The following supporting information can be downloaded at https://www.mdpi.com/article/10.3390/jcm15030973/s1: Table S1: PRISMA 2020 checklist; Table S2: Commercially available root canal sealers used in the studies included in this systematic review; Table S3: Results of risk of bias assessment of in vitro studies according to the guidelines for reporting of preclinical studies on dental materials by Faggion Jr. [26].
