# Supplementary material for: Cytotoxicity of Root Canal Sealers and Potential Clinical Implications: A Comprehensive Systematic Review of In Vitro Studies
Source: J Clin Med. 2026 Jan 25;15(3):973. doi: 10.3390/jcm15030973 (PMC12897702; doi:10.3390/jcm15030973)
Supplement: Supplementary file 1 [file jcm-15-00973-s001.zip › jcm-4099846-supplementary.pdf]

Journal of Clinical Medicine

MDPI

Supplementary Materials

Cytotoxicity of Root Canal Sealers and Potential Clinical Implications: A Comprehensive Systematic Review of In Vitro Studies

Mirko Piscopo[1,†], Angelo Aliberti[1,†*], Roberta Gasparro[1], Gilberto Sammartino[1*], Noemi Coppola[1], Pietro Ausiello[1]

[1] Department of Neurosciences, Reproductive and Odontostomatological Sciences, University of Naples Federico II, Naples, Italy; mirk.piscopo@studenti.unina.it (M.P.); ange.aliberti@studenti.unina.it (A.A.) roberta.gasparro@unina.it R.G.); gilberto.sammartino@unina.it (G.S.); noemi.coppola@unina.it (N.C.); pietausi@unina.it (P.A.)

* Correspondence: ange.aliberti@studenti.unina.it; gilberto.sammartino@unina.it

† These authors contributed equally to the work.

Table S1: PRISMA 2020 checklist

Section and Topic	Item #	Checklist item	Reported (Yes/No)
**TITLE**			
Title	1	Identify the report as a systematic review.	Y
**BACKGROUND**			
Objectives	2	Provide an explicit statement of the main objective(s) or question(s) the review addresses.	Y
**METHODS**			
Eligibility criteria	3	Specify the inclusion and exclusion criteria for the review.	Y
Information sources	4	Specify the information sources (e.g. databases, registers) used to identify studies and the date when each was last searched.	Y
Risk of bias	5	Specify the methods used to assess risk of bias in the included studies.	Y
Synthesis of results	6	Specify the methods used to present and synthesise results.	Y

Section and Topic	Item #	Checklist item	Reported (Yes/No)
**RESULTS**			
Included studies	7	Give the total number of included studies and participants and summarise relevant characteristics of studies.	Y
Synthesis of results	8	Present results for main outcomes, preferably indicating the number of included studies and participants for each. If meta-analysis was done, report the summary estimate and confidence/credible interval. If comparing groups, indicate the direction of the effect (i.e. which group is favoured).	Y
**DISCUSSION**			
Limitations of evidence	9	Provide a brief summary of the limitations of the evidence included in the review (e.g. study risk of bias, in-consistency and imprecision).	Y
Interpretation	10	Provide a general interpretation of the results and important implications.	Y
**OTHER**			
Funding	11	Specify the primary source of funding for the review.	Y
Registration	12	Provide the register name and registration number.	Y

Journal of
Clinical Medicine

Table S2. Commercially available root canal sealers used in the studies included in this systematic review.

Type	Sealer	Manufacturer	Study
**ZnO-eugenol**	Pupl Canal Sealer	Kerr, Romulus, USA	[45,61,62,69,104,107,111,116]
	Pupl Canal Sealer Extended Working Time	Kerr, Romulus, USA	[80,94,96,99]
	N2	Indrag-Agsa, Losone, Switzerland	[101,108,109,119]
	Endofill	Produits Dentaires, Vevey Switzerland	[45,75,92,112]
	Canals	Showa Pharmaceutical Co., Tokyo, Japan	[101,108,109,120]
	Endométhasone	Septodont, Saint-Maur-des-Fossés, France	[55,80,119,123]
	Roth's Sealer	Roth International, Chicago, USA	[72,93]
	Zinc Oxide-Eugenol	Produits Dentaires, Vevey Switzerland	[121]
	Tubli-Seal	Kerr, Romulus, USA	[71,74]
	Tubli-Seal Xpress	Kerr, Romulus, USA	[97]
	Pulpdent Root Canal Sealer	Pulpdent Corp. Watertown, U.S.A.	[53]
	FillCanal	Technew, Rio de Janeiro, RJ, Brasil	[84,105]
**Resin (epoxy)**	AH Plus	Dentsply DeTrey Gmbh, Konstanz, Germany	[28,29,32,37,40,41,43,44,48,50,51,52, 53,54,57,60,62,63,64,65,66,67,69,72, 74,75,76,79,80,81,82,85,88,90,91,93, 96,97,98,100,103,105,110,112,113,11 4,115,117,118,119,120,121,122]
	AH 26	Dentsply DeTrey Gmbh, Konstanz, Germany	[99,101,102,106,108,109,118,120,121 ,122,123]
	AH Plus Jet	Dentsply DeTrey Gmbh, Konstanz, Germany	[31,35,36,41,68,70,71,87,89,95,111]
	Acroseal	Septodont, Saint-Maur-des-Fossés, France	[82,113]
	SimpliSeal	Discuss Dental LLC, Calver City, USA	[73,75]
	TopSeal	Dentsply DeTrey Gmbh, Konstanz, Germany	[116]
	Sealer Plus	MK Life, Porto Alegre, Brazil	[75]
	ThermaSeal Plus	Dentsply/Maillefer, Konstanz, Germany	[93]
	Dia-Proseal	DiaDent Group, Cheongju-si, Korea.	[53]

	AD Seal	Meta Biomed, Cheongju, Korea	[64]
**Resin (methacrylate)**	EndoREZ	Ultradent, South Jordan, USA	[70,74,85,95,98,104,110,113]
	Epiphany	Pentron, Wallingford, USA	[98,107,110,113,114]
	Epiphany SE	Pentron, Wallingford, USA	[92,107]
	RealSeal	SybronEndo, Orange, USA	[55,70,88,95,106]
	RealSeal SE	SybronEndo, Orange, USA	[93,104]
	RealSeal XT	SybronEndo, Orange, USA	[87]
	MetaSEAL	Parkell, Inc., Farmington, USA	[98,104,111]
	MetaSEAL Soft	Sun Medical, Tokyo, Japan	[71]
	SuperBond sealer	Sun Medical, Shiga, Japan	[49]
**Glass ionomer**	Ketac Endo	3M ESPE, St. Paul, USA	[119,123,124]
	Activ GP	Brasseler, Savannah, USA	[106]
	Nishika Canal Sealer BG	Nippon Shika Yakuhin, Yamaguchi, Japan	[49]
	Endion	VOCO, Germany	[124]
**Silicone**	GuttaFlow	Roeko/Coltène/Whaledent, Langenau, Germany	[71,93,97,100,113]
	GuttaFlow 2	Roeko/Coltène/Whaledent, Langenau, Germany	[49,51,53,66,72,76,88]
	GuttaFlow Bioseal	Roeko/Coltène/Whaledent, Langenau, Germany	[50,66,76]
	RoekoSeal	Roeko/Coltène/Whaledent, Langenau, Germany	[85,113]
	RoekoSeal Automix	Roeko/Coltène/Whaledent, Langenau, Germany	[55,110,115,116]
**Calcium hydroxide**	Sealapex	Kerr, Romulus, USA	[37,55,74,86,93,117,120]
	Apexit	Ivoclar Vivadent, Schaan, Liechtenstein	[70,113,119]
	Apexit Plus	Ivoclar Vivadent, Schaan, Liechtenstein	[37,71]
	Sealer 26	Dentsply/Maillefer, Konstanz, Germany	[45,122]
	L&C	Dentsply Herpo, Petrópolis, RJ, Brazil	[105]
	Calciobiotic Root Canal Sealer	Hygenic, Akron, Ohio, USA	[123]
**Bioactive**	MTA Fillapex	Angelus, Londrina, Brazil	[33,37,46,56,62,63,66,69,71,72,73,76,79,80,81,82,84,86,89,91,92]
	MTA Angelus	Angelus, Londrina, Brazil	[30,91]
	i-MTA	Longly Biotechnology, Wuhan, China	[27]
	Endoseal MTA	Maruchi, Seoul, Korea	[59,64,67,77]

AGM MTA	AGM, Tehran, Iran	[30]
NEOMTA Plus	Avalon biomed Houston, TX, USA	[33]
BrightEndo MTA	Dentium Co., Seoul, Republic of Korea	[54]
ProRoot MTA	Dentsply Tulsa Dental, Tulsa, OK, USA	[78,88]
MTA Plus	Avalon Biomed Inc., Bradenton, FL, USA	[84]
Apatite Root Sealer	Sankin Kogyo, Tokyo, Japan	[86,94,99,123]
BioRoot RCS	Septodont, Saint-Maur-des-Fossés, France	[36,42,50,61,62,67,68,69,72,73,77,83]
Endosequence BC	Brasseler, Savannah, USA	[35,47,48,58,59,60,63,67,68,72,96,97]
EndoSequence BC HIFlow	Brasseler, Savannah, USA	[31,44,58,60]
iRoot SP	Innovative BioCeramix Inc., Vancouver, Canada	[27,29,34,40,44,52,86,89,103]
iRoot BP Plus	Innovative BioCeramix Inc., Vancouver, Canada	[78]
iRoot FS	Innovative BioCeramix Inc., Vancouver, Canada	[78]
CeraSeal	MetaBiomed, Cheongju, Korea	[28,43,54,59]
Neosealer flo	Avalon biomed Houston, TX, USA	[27,28,34,38]
Bio-C sealer	Angelus, Londrina, Brazil	[31,32,33,42,46,.47,.56.65]
Bio-C Sealer ion +	Angelus, Londrina, Brazil	[32]
AH Plus Bioceramic	Dentsply DeTrey, Konstanz, Germany	[29,33,35,36,38,39,41,48]
BioRoot Flow	Septodont, Saint-Maur-des-Fossés, France	[36,39]
TotalFill BC	FKG Dentaire, La-Chaux-de-Fonds, SwitzerlandMTA	[37,38,41,50,57,65,79]
CRoot SP	Beijing C-Root Dental Medical Devices Co. Ltd, China	[27,29,40]
Sealer Plus bioceramic	MK Life, Porto Alegre, Brazil	[42,47,57]
Cimmo HP	Cimmo Soluções em Saùde, Pouso Alegre, MG, Brazil	[46,56]
Endoseal TCS	MARUCHI, Wonju, Korea	[54]
Nano-Ceramic Sealer	B&L Biotech, Fairfax, VA, USA	[64,77]
Wellroot ST	Vericom, Chuncheon, South Korea	[64]

Table S3. Results of risk of bias assessment of in vitro studies according to the guidelines for reporting of preclinical studies on dental materials by Faggion Jr. [26].

	**Checklist item**														
**Study**	**1**	**2a**	**2b**	**3**	**4**	**5**	**6**	**7**	**8**	**9**	**10**	**11**	**12**	**13**	**14**
**Chen Z. et al.[27] 2025**	Y	Y	Y	Y	Y	N	N	N	N	Y	Y	Y	Y	Y	N
**Gaafar, S. S. et al.[28] 2025**	Y	Y	Y	Y	Y	N	N	N	N	Y	Y	Y	Y	Y	N
**Kwan D.C.Y. et al.[29] 2025**	Y	Y	Y	Y	Y	N	N	N	N	Y	Y	Y	Y	N	N
**Ramos R.F. et al.[30] 2025**	Y	Y	Y	Y	Y	N	N	N	N	N	Y	Y	Y	Y	N
**Wang Z. et al.[31] 2025**	Y	Y	Y	Y	Y	N	N	N	N	Y	Y	Y	Y	Y	N
**Ye Y. et al.[32] 2025**	Y	Y	Y	Y	Y	N	N	N	N	Y	Y	Y	Y	N	N
**Nashibi S. et al.[33] 2025**	Y	Y	Y	Y	N	N	N	N	N	Y	Y	Y	N	N	N
**Pitzschk M.A.L.R. et al.[34] 2025**	Y	Y	Y	Y	Y	N	N	N	N	Y	Y	Y	Y	N	N
**Santiago M.C. et al.[35] 2025**	Y	Y	Y	Y	Y	N	N	N	N	Y	Y	Y	Y	N	N
**Chen J.H. et al.[36] 2024**	Y	Y	Y	Y	Y	Y	N	N	N	N	Y	Y	N	N	N
**Çelebi Keskin İ.S. et al.[37] 2024**	N	Y	Y	Y	Y	N	N	N	N	N	Y	Y	Y	N	N
**López-García S. et al.[38] 2024**	Y	Y	Y	Y	Y	N	N	N	N	N	Y	Y	Y	Y	N
**Sanz J.L. et al.[39] 2024**	Y	Y	Y	Y	Y	Y	N	N	N	N	Y	Y	Y	Y	Y
**Zhou G. et al.[40] 2024**	Y	Y	Y	Y	Y	N	N	N	N	N	Y	Y	Y	Y	N
**Kandemir Demirci G. et al.[41] 2023**	Y	Y	Y	Y	Y	Y	N	N	N	Y	Y	Y	Y	N	N
**Melo A.P. et al.[42] 2023**	Y	Y	Y	Y	Y	Y	N	N	N	N	Y	Y	Y	Y	N
**Nguyen L.C.H. et al.[43] 2023**	N	Y	Y	Y	Y	N	N	N	N	N	Y	Y	Y	N	N
**Yan Y. et al.[44] 2023**	Y	Y	Y	Y	Y	Y	N	N	N	N	Y	Y	Y	Y	N
**Oliveira P.Y. et al.[45] 2022**	Y	Y	Y	Y	Y	N	N	N	N	N	Y	Y	Y	N	N
**Pedrosa M.D.S. et al.[46] 2022**	Y	Y	Y	Y	Y	N	N	N	N	N	Y	Y	Y	N	N
**Rosatto C.M.P. et al.[47] 2022**	Y	Y	Y	Y	Y	Y	N	N	N	N	Y	Y	Y	N	N
**Sanz J.L. et al.[48] 2022**	Y	Y	Y	Y	Y	Y	N	N	N	N	Y	Y	Y	Y	N
**Tomokiyo A. et al.[49] 2022**	Y	Y	Y	Y	Y	Y	N	N	N	N	Y	Y	Y	Y	N

**Wuersching S.N. et al.[50] 2022**	Y	Y	Y	Y	Y	Y	N	N	N	N	Y	Y	Y	Y	N
**Dhopavkar V.V. et al.[51] 2021**	Y	Y	Y	Y	Y	N	N	N	N	N	Y	Y	Y	Y	N
**Erdogan H. et al.[52] 2021**	Y	Y	Y	Y	Y	Y	N	N	N	N	Y	Y	Y	N	N
**Khan M.T. et al.[53] 2021**	Y	Y	Y	Y	Y	Y	N	N	N	N	Y	Y	Y	Y	N
**Park M.G. et al.[54] 2021**	Y	Y	Y	Y	Y	N	N	N	N	N	Y	Y	Y	N	N
**Pawinska M. et al.[55] 2021**	Y	Y	Y	Y	Y	Y	N	N	N	N	Y	Y	Y	N	N
**Pedrosa M.D.S. et al.[56] 2021**	Y	Y	Y	Y	Y	N	N	N	N	N	Y	Y	Y	N	N
**Zordan-Bronzel C.L. et al.[57] 2021**	Y	Y	Y	Y	Y	Y	N	N	N	N	Y	Y	Y	N	N
**Chen B. et al.[58] 2020**	Y	Y	Y	Y	Y	Y	N	N	N	N	Y	Y	Y	N	N
**López-García S. et al.[59] 2020**	Y	Y	Y	Y	Y	Y	N	N	N	N	Y	Y	Y	Y	N
**Rodríguez-Lozano F.J. et al.[60] 2020**	Y	Y	Y	Y	Y	Y	N	N	N	N	Y	Y	Y	Y	N
**Jeanneau C. et al.[61] 2019**	Y	Y	Y	Y	Y	N	N	N	N	Y	Y	Y	Y	Y	N
**Jung S. et al.[62] 2019**	Y	Y	Y	Y	N	N	N	N	N	Y	Y	Y	Y	N	N
**Lee B.N. et al.[63] 2019**	Y	Y	Y	Y	Y	Y	N	N	N	N	Y	Y	Y	N	N
**Lee J.K. et al.[64] 2019**	Y	Y	Y	Y	Y	Y	N	N	N	N	Y	Y	Y	Y	N
**López-García S. et al.[65] 2019**	Y	Y	Y	Y	Y	N	N	N	N	N	Y	Y	Y	Y	N
**Rodríguez-Lozano F.J. et al.[66] 2019**	Y	Y	Y	Y	Y	Y	N	N	N	N	Y	Y	Y	Y	N
**Seo D. G. et al.[67] 2019**	Y	Y	Y	Y	Y	N	N	N	N	Y	Y	Y	Y	N	N
**Alsubait S.A. et al.[68] 2018**	Y	Y	Y	Y	Y	Y	N	N	N	N	Y	Y	Y	N	N
**Jung S. et al.[69] 2018**	Y	Y	Y	Y	Y	Y	N	N	N	N	Y	Y	Y	Y	N
**Martinho F.C. et al.[70] 2018**	Y	Y	Y	Y	Y	Y	N	N	N	N	Y	Y	Y	Y	N
**Szczurko G. et al.[71] 2018**	Y	Y	Y	Y	Y	N	N	N	N	Y	Y	Y	Y	N	N
**Taraslia V. et al.[72] 2018**	Y	Y	Y	Y	Y	N	N	N	N	N	Y	Y	Y	N	N
**Vouzara T. et al.[73] 2018**	Y	Y	Y	Y	Y	N	N	N	N	N	Y	N	Y	N	N
**Arun S. et al.[74] 2017**	Y	Y	Y	Y	N	N	N	N	N	Y	Y	Y	N	N	N
**Cintra L.T.A. et al.[75] 2017**	Y	Y	Y	Y	Y	N	N	N	N	N	Y	Y	Y	N	N
**Collado-González M. et al.[76] 2017**	Y	Y	Y	Y	Y	Y	N	N	N	N	Y	Y	Y	Y	N

Collado-González M. et al.[77] 2017	Y	Y	Y	Y	Y	Y	N	N	N	N	Y	Y	Y	Y	N
Lv F. et al.[78] 2017	Y	Y	Y	Y	Y	Y	N	N	N	N	Y	Y	Y	N	N
Rodríguez-Lozano F.J. et al.[79] 2017	Y	Y	Y	Y	Y	N	N	N	N	N	Y	Y	Y	N	N
Silva E.J. et al.[80] 2016	N	Y	Y	Y	Y	N	N	N	N	N	Y	Y	Y	N	N
Silva E.J. et al.[81] 2016	N	Y	Y	Y	Y	N	N	N	N	N	Y	Y	Y	Y	N
Suciu I. et al.[82] 2016	Y	Y	Y	Y	N	N	N	N	N	Y	Y	Y	Y	N	N
Dimitrova-Nakov S. et al.[83] 2015	Y	Y	Y	Y	Y	Y	N	N	N	N	U	Y	N	N	N
Mestieri L.B. et al.[84] 2015	Y	Y	Y	Y	Y	Y	N	N	N	N	Y	Y	U	N	N
Camargo C.H. et al.[85] 2014	Y	Y	Y	Y	Y	Y	N	N	N	N	Y	Y	Y	N	N
Chang S.W. et al.[86] 2014	Y	Y	Y	Y	Y	Y	N	N	N	N	Y	Y	Y	N	N
Cotti E. et al.[87] 2014	Y	Y	Y	N	N	N	N	N	N	Y	Y	Y	Y	N	N
Manda P. et al.[88] 2014	Y	Y	Y	Y	N	N	N	N	N	Y	Y	Y	Y	Y	N
Güven E.P. et al.[89] 2013	Y	Y	Y	Y	Y	Y	N	N	N	N	Y	Y	U	N	N
Kim T.G. et al.[90] 2013	Y	Y	Y	Y	N	N	N	N	N	Y	Y	Y	N	N	N
Bin C.V. et al.[91] 2012	Y	Y	Y	Y	Y	Y	N	N	N	N	Y	Y	N	N	N
Salles L.P. et al.[92] 2012	Y	Y	Y	Y	Y	Y	N	N	N	N	Y	Y	N	N	N
Scelza M.Z. et al.[93] 2012	Y	Y	Y	Y	Y	Y	N	N	N	N	Y	U	N	N	N
Shon W.J. et al.[94] 2012	Y	Y	Y	Y	Y	Y	N	N	N	N	Y	U	N	N	N
Van Landuyt K.L. et al.[95] 2012	Y	Y	Y	Y	N	N	N	N	N	N	Y	Y	Y	N	N
Loushine B.A. et al.[96] 2011	Y	Y	Y	Y	Y	Y	N	N	N	N	Y	Y	Y	N	N
Zoufan K. et al.[97] 2011	Y	Y	Y	Y	Y	Y	N	N	N	N	N	Y	N	N	N
Al-Hiyasat A.S. et al.[98] 2010	Y	Y	Y	Y	Y	Y	N	N	N	N	Y	Y	Y	Y	N
Bae W.J. et al.[99] 2010	Y	Y	Y	Y	Y	Y	N	N	N	N	Y	Y	Y	N	N
Ghanaati S. et al.[100] 2010	N	Y	Y	Y	Y	N	N	N	N	N	Y	U	U	N	N
Huang F.M. et al.[101] 2010	Y	Y	Y	Y	N	N	N	N	N	N	Y	Y	Y	N	N
Yu M.K. et al.[102] 2010	Y	Y	Y	Y	N	N	N	N	N	Y	Y	Y	Y	N	N
Zhang W. et al.[103] 2010	Y	Y	Y	Y	N	N	N	N	N	Y	Y	Y	Y	N	N
Ames J.M. et al.[104] 2009	Y	Y	Y	Y	N	N	N	N	N	Y	Y	Y	Y	N	N
Correa G.T. et al.[105] 2009	Y	Y	Y	Y	Y	N	N	N	N	N	Y	Y	U	N	N

**Donadio M. et al.[106] 2009**	Y	Y	Y	Y	U	U	N	N	N	N	Y	Y	U	N	N
**Gambarini G. et al.[107] 2009**	Y	Y	Y	Y	Y	N	N	N	N	N	Y	Y	N	N	N
**Huang F.M. et al.[108] 2009**	Y	Y	Y	Y	N	N	N	N	N	Y	Y	Y	Y	N	N
**Huang F.M. et al.[109] 2008**	Y	Y	Y	Y	N	N	N	N	N	Y	Y	Y	Y	N	N
**Lodiene G. et al.[110] 2008**	Y	Y	Y	Y	Y	Y	N	N	N	N	Y	Y	U	N	N
**Pinna L. et al.[111] 2008**	N	Y	Y	Y	Y	N	N	N	N	N	Y	Y	Y	N	N
**Valois C.R. et al.[112] 2008**	Y	Y	Y	Y	Y	Y	N	N	N	N	Y	Y	Y	N	N
**Eldeniz A.U. et al.[113] 2007**	Y	Y	Y	Y	Y	Y	N	N	N	N	Y	Y	Y	N	N
**Merdad K. et al.[114] 2007**	N	Y	Y	Y	Y	N	N	N	N	N	Y	N	N	N	N
**Miletić I. et al.[115] 2005**	N	Y	Y	Y	Y	N	N	N	N	N	Y	Y	N	N	N
**Bouillaguet S. et al.[116] 2004**	N	Y	Y	Y	Y	Y	N	N	N	N	Y	Y	Y	Y	N
**Huang T.H. et al.[117] 2004**	N	Y	Y	Y	Y	N	N	N	N	N	Y	Y	N	N	N
**Huang F.M. et al.[118] 2002**	Y	Y	Y	Y	Y	Y	N	N	N	N	Y	Y	Y	N	N
**Schwarze T. et al.[119] 2002**	N	Y	Y	Y	Y	N	N	N	N	N	Y	U	U	N	N
**Hunag T.H. et al.[120] 2001**	N	Y	Y	Y	Y	Y	N	N	N	N	Y	Y	Y	N	N
**Azar N.G. et al.[121] 2000**	N	Y	Y	Y	Y	N	N	N	N	N	Y	N	N	N	N
**Huang T.H. et al.[122] 2000**	Y	Y	Y	Y	Y	Y	N	N	N	N	Y	Y	Y	N	N
**Telli C. et al.[123] 1999**	N	Y	Y	Y	Y	N	N	N	N	N	Y	U	U	N	N
**Beltes P. et al.[124] 1997**	N	Y	Y	Y	Y	N	N	N	N	N	Y	N	N	N	N

Abbreviations: N, No; Y, Yes; U, Unclear. Checklist items: 1- Structured abstract; 2a – Scientific background; 2b - Research aims and/or hypotheses; 3 - Description of the intervention for each group; 4 - Definition of the measured outcomes; 5 -Sample size Determination; 6 - Method used to create the allocation sequence; 7 - Procedure for concealment of the allocation; 8 - Implementation; 9 - Blinding procedure; 10 - Statistical approaches used; 11 - Outcome results and effect estimates: 12 – Limitations; 13 – Funding information; 14 - Availability of the study protocol (if provided).
